# Close encounters of the friendly kind: pacific between‐group interactions in primates

**DOI:** 10.1111/brv.70046

**Published:** 2025-06-16

**Authors:** Cyril C. Grueter, Luca Pozzi

**Affiliations:** ^1^ Department of Anatomy, Physiology and Human Biology, School of Human Sciences The University of Western Australia 35 Stirling Highway Perth Western Australia 6009 Australia; ^2^ International Centre for Biodiversity and Primate Conservation, Dali University 2 Hongsheng Road Dali Yunnan 671003 China; ^3^ Centre for Evolutionary Biology, School of Biological Sciences The University of Western Australia 35 Stirling Highway Perth Western Australia 6009 Australia; ^4^ Centre of Excellence in Biodiversity and Natural Resource Management University of Rwanda KN 67 Street, Nyarugenge, P.O. Box 3900 Kigali Rwanda; ^5^ School of Anthropology and Museum Ethnography University of Oxford 51–53 Banbury Road Oxford OX2 6PE UK; ^6^ Department of Anthropology The University of Texas at San Antonio One UTSA Circle San Antonio Texas 78249‐1644 USA

**Keywords:** intergroup encounters, intergroup tolerance, intergroup affiliation, intergroup coalitions, intergroup mingling, group merger, multilevel society, primates

## Abstract

While intergroup conflict features prominently in the behavioural ecology literature, its antonym, intergroup peace, has been a rather neglected phenomenon until recently. Neighbourly relations and affiliative interactions are far from uncommon. We provide a comprehensive synthesis of the empirical evidence of peaceful between‐group interactions in primates which take various forms that vary along two continua: intensity and duration. These include intergroup tolerance, intergroup affiliation, visits, mingling, intergroup coalitions and mergers between groups. We propose that an analysis of intergroup peace would benefit from distinguishing between facilitating factors (facilitators), active drivers and stabilizing forces (stabilizers). A prime example of a facilitator is resource heterogeneity in the landscape which can make resource defence logistically impractical and make initially independent groups more interdependent. While this often suffices for tolerant associations to emerge, for full‐blown intergroup peace with sporadic or frequent intergroup affiliation and/or cooperation to arise, motivations or adaptive benefits (drivers) need to be present. Some of the adaptive benefits that can trigger a switch from tolerance to peace between groups include dispersal facilitation, information gathering, predation protection, extra‐group mating, communal defence, and reciprocal resource access. Lastly, a further mediator of xenophobia and intergroup agonism is the degree of relatedness and familiarity between interacting groups (stabilizers). Finally, we propose several ways to move this research forward. We hope that this review will stimulate empirical and theoretical studies and encourage field researchers to pay more attention to hitherto rather neglected forms of between‐group contact. An understanding of the functional correlates of peaceful between‐group relationships in primates also holds promise for making inferences about the human social system where intergroup peace has enabled cooperation and cultural diffusion on an unprecedented scale.

## I. INTRODUCTION

In non‐human primates and other animals, relationships between social units are often characterized by enmity driven by mating or resource competition (Fashing, 2001; Brown, 2013; Van Belle, Grueter & Furuichi, 2020). The factors responsible for escalation of intergroup interactions have been explored in a series of studies (Wilson *et al*., 2012; Roth & Cords, 2016; De Dreu & Triki, 2022) and fall into two major categories: the difference in the combined competitive ability or resource‐holding potential of interacting groups (which is often a function of group size and possibly group cohesion) (Green, Briffa & Cant, 2021) and the potential gains to be made from winning an encounter (i.e. the payoffs in terms of acquisition of contested resources) (Parker, 1974; Cheney, 1987; Kitchen & Beehner, 2007; Arnott & Elwood, 2009).

While animosity is often seen as the default in the context of intergroup interactions, there is extensive variation among and within species. Much less emphasis has been placed on the factors underlying non‐agonistic encounters although research interest is increasing (Robinson & Barker, 2017; Pisor & Surbeck, 2019). Following the Hawk–Dove model (Maynard Smith & Price, 1973), a non‐aggressive (Dove) strategy should prevail when the costs of aggression outweigh its benefits. Under favourable conditions, such as reduced resource competition, intergroup interactions may shift towards mutual tolerance, peaceful encounters, and even temporary mingling. Such amicable interactions across group boundaries have been recorded in organisms as diverse as ants (Robinson, 2014), fiddler crabs (Backwell & Jennions, 2004), birds (e.g. Hale, Williams & Rabenold, 2003), foxes (Murdoch *et al*., 2008) and dolphins (Connor *et al*., 2022). Among primates, these are particularly prevalent in the apes, e.g. western gorillas (*Gorilla gorilla*), mountain gorillas (*Gorilla beringei*), bonobos (*Pan paniscus*), and white‐handed gibbons (*Hylobates lar*) (reviewed in Grueter & Wilson, 2021) but also in various monkey species (see below). In primate species with multilevel societies, core social units are in a permanent state of association with other units (Grueter, Chapais & Zinner, 2012a; Grueter *et al*., 2020). Multilevel societies have been confirmed to exist some in colobine monkeys, papionins, humans, a few non‐primate mammals and some birds (reviewed in Grueter *et al*., 2020). An extreme manifestation of intergroup peace is enduring group mergers (e.g. Danaher‐Garcia *et al*., 2022).

Little is known, however, about the machinery creating “primate peace systems”. Most accounts of peaceful interactions and relationships are descriptive, with a functionalist angle largely missing (but see Mirville *et al*., 2018b; Lucchesi *et al*., 2020). Tolerant and affiliative intergroup encounters may well be fitness‐enhancing for the participants and may have been positively selected to increase in temporal continuity and to recur (Furuichi, 2020; Pisor & Surbeck, 2019). For example, successful extra‐group copulation can lead to positive reproductive outcomes for the individual engaged in this behaviour. Or, cooperative interactions can indirectly increase reproductive success *via* access to contested resources, as seen in between‐group (“third‐order”) alliances in the case of bottlenose dolphins (*Tursiops* sp.) (Connor *et al*., 2022). In Seychelles warblers (*Acrocephalus sechellensis*), male territory owners who live next to related or familiar males (with whom they were on relatively friendly terms) gained body mass (by not having to invest too much energy in defending territory borders) and showed less telomere attrition (Bebbington *et al*., 2017).

Here, we present a three‐pronged framework for predicting the occurrence and likelihood of peaceful interactions between groups. We define groups as relatively stable spatial associations of conspecific (and occasionally heterospecific) individuals which interact with one other more than with other individuals in the vicinity (Kummer, 1971; Pisor & Surbeck, 2019). In this review, we define the core unit (*sensu* Grueter *et al*., 2020) as the group in multilevel societies, given its stability, cohesion, and strong social bonds. However, we acknowledge that the upper level (e.g. the band) could also be considered a group. The prevalence of pacific interactions in between‐group dynamics depends on three key elements or supporting pillars: facilitators, drivers, and stabilizers (Table 1). Facilitators impact the cost side of peaceful interactions by reducing interaction costs, thereby increasing the likelihood of peaceful encounters. These include cognitive capacities, socio‐demographic features, pathogen prevalence, and landscape features. For example, resource distribution that promotes contact between social units can foster peaceful interactions. Drivers represent the direct benefits of peaceful interactions, such as joint resource and mate defence, extra‐group mating, dispersal facilitation, and reciprocal resource access. Stabilizers are the underlying principles that maintain or enhance the benefits of peaceful associations, such as familiarity between groups or members, and indirect fitness benefits through kin selection. When all three elements – facilitators, drivers, and stabilizers – are present, we expect peaceful interactions to become more prevalent and exhibit higher levels of stability.

**Table 1:** Facilitators, drivers and stabilizers of peaceful intergroup interactions and relationships.

Facilitators	Drivers	Stabilizers
Cognitive capacities Group size and composition Male coalitions Shared decision making Population density Reproductive status of females Interaction location Arboreality Low pathogen prevalence Diet and resource distribution (e.g. resource heterogeneity) By‐product of within‐group affiliation	Dispersal facilitation Information gathering (pre‐dispersal prospecting, assessment of competitive ability) Protection from predators Facilitation of resource acquisition Reciprocal resource access Joint resource defence Collective/communal mate defence Extra‐group mating	Relatedness Familiarity

**Fig. 1:**
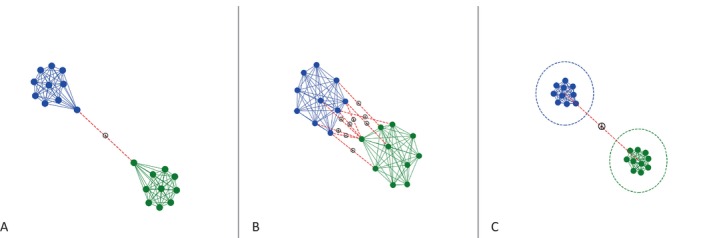
Networks illustrating the distinctions among peaceful intergroup interactions involving single individuals (A), subsets of individuals (B), and entire groups (C).

Moreover, the benefits of peaceful interactions can accrue to individuals, subsets of individuals, or all members of the group (Fig. 1). While individual strategies may be constrained by factors such as phylogeny, it is valuable to view individual behaviour as a flexible, optimal response to socioecological conditions (Davies, Krebs & West, 2012; Jaeggi *et al*., 2016). Conflicts of interest can arise when optimal strategies among individuals clash. For instance, in species where males disperse, subadult males might seek affiliative intergroup interactions to explore dispersal opportunities, while females might focus on defending group interests. Similarly, a female might pursue extra‐group copulations for potential reproductive benefits, whereas males might resist these actions to protect their reproductive assets. Or, juveniles may gain valuable benefits, such as improved motor skills, from playing with extra‐group peers, while adults may face increased costs due to the heightened potential for conflict when near members of another group. When interests align, the benefits can extend to all group members, such as through collective defence against predators or shared rivals. However, in cases of collective defence against usurping outsider males, some females might benefit from the good genes of the usurper, even if he engages in infanticide.

In this review, we begin by identifying six forms of intergroup peace, then turn to reviewing the facilitators, drivers, and stabilizers of intergroup peace. In discussing these we highlight the cost–benefit structure and potential conflicts of interest among individuals wherever applicable. We provide case examples of these concepts throughout, drawing from the non‐human primate literature. We do, however, where appropriate, throw in the occasional non‐primate citation to illustrate the generality of some of our arguments.

## II. DISTRIBUTION AND FORMS OF PACIFIC INTERGROUP INTERACTIONS

**Fig. 2:**
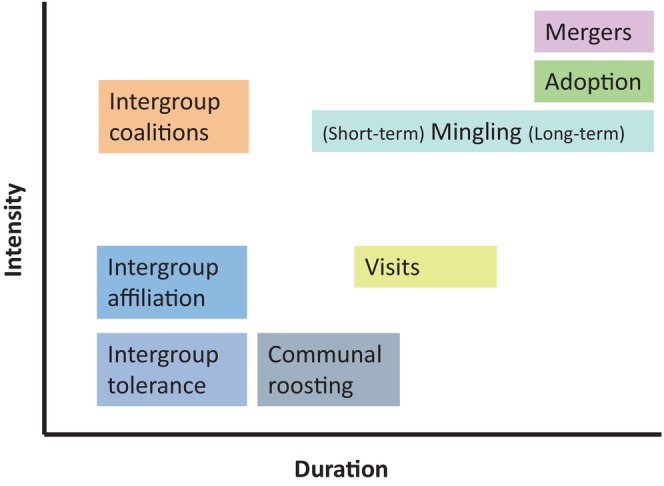
Forms of pacific interactions in primates that vary in terms of duration and intensity. Mingling (monospecific associations) can range from relatively short‐lived (hours to days) as in species with supra‐level organization to long‐lasting/permanent (years), as in species with multilevel systems (see text for details).

Intergroup peace is an umbrella term that encompasses various non‐agonistic interactions that vary along two axes: intensity (i.e. level of social involvement) and duration. Here we identify eight forms (Table 2; Fig. 2). The six most common forms are: mutual tolerance when two groups come into proximity (e.g. when simultaneously exploiting a resource); affiliation, that is the exchange of socio‐positive behaviours between individuals from different groups during group encounters; visits, that is temporary residence in another group; mingling, that is protracted tolerant encounters between groups that can also include affiliative elements; coalitions, that is joint between‐group action against a third party; and mergers, that is the long‐lasting or permanent fusion of two groups into a new cohesive entity. Two additional, rarer forms of intergroup peace are communal roosting and intergroup adoption. The categories subsumed under intergroup peace are not mutually exclusive and can co‐occur in the same species, for example mingling provides an arena for affiliative or cooperative interactions.

**Table 2:** Definitions and examples of six types of pacific intergroup interactions.

Phenomenon	Definition	Example
Tolerance	Indifference and absence of agonism when individuals from two groups come into proximity	Simultaneous exploitation of a resource by two groups
Affiliation	Exchange of socio‐positive behaviours between one or more individuals of different groups when groups are in proximity	Grooming, playing and food sharing during intergroup encounters
Visits	Temporary residence in another group	Individuals entering a neighbouring group and remaining with it for up to several days
Mingling	Protracted encounter between groups (mostly conspecific, but also heterospecific) that can also include affiliative elements and is often characterized by coordinated activities	Short‐term associations (lasting from a few hours to a few days) and long‐term associations (multilevel societies)
Cooperation/coalitions	Transfer of benefits from one group to another, resulting in net benefits shared by multiple members of the groups involved	Joint between‐group defence against a third party (e.g. conspecific bachelor male or predator)
Mergers	Partial or complete fusion of hitherto separate groups into a new cohesive entity	Assimilation of two separate dolphin communities into a single community

**Fig. 3:**
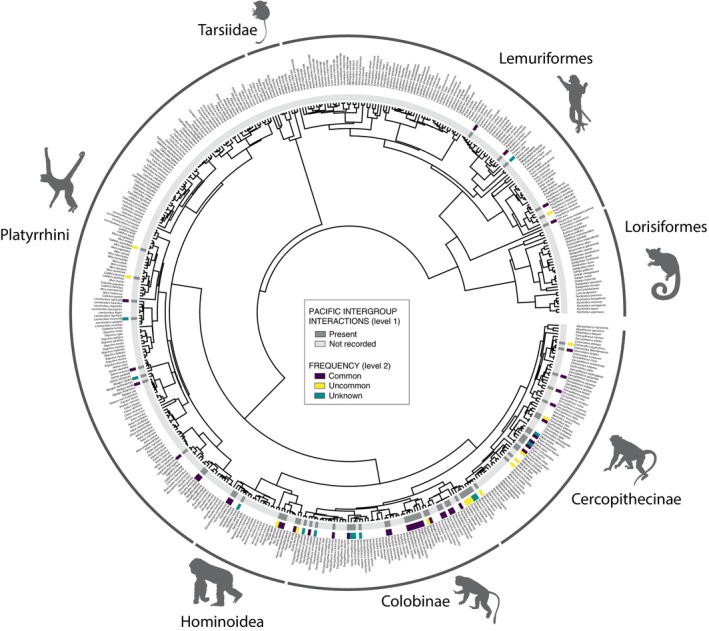
Phylogram of extant primates depicting all species in which tolerant/non‐agonistic and affiliative intergroup interactions (including short‐term mingling) have been recorded. The phylogram also indicates whether such interactions are common or uncommon for species for which this information is available. Tree topology was obtained from Upham *et al*. (2019) and primate silhouettes from https://www.phylopic.org/. Data sources can be found in Table S1.

Pacific intergroup interactions have been recorded in primate species representing all major radiations, that is American monkeys (platyrrhines), Afro‐Eurasian monkeys and apes (catarrhines), and lemurs and lorises (strepsirrhines) (Fig. 3, see online Supporting Information, Table S1, for data sources). Tolerant and affiliative intergroup interactions have been reported for 68 primate species, both territorial and non‐territorial. Based on the currently available field evidence, there are 22 species where the predominant mode of intergroup interaction is pacific. These include all the species featuring multilevel societies plus the following species with single‐level societies: red‐handed howler monkey (*Alouatta belzebul*), red‐tailed monkey (*Cercopithecus ascanius*), De Brazza's monkey (*Cercopithecus neglectus*), tantalus monkey (*Chlorocebus tantalus*), western gorilla, bonobo, Tana River red colobus (*Piliocolobus rufomitratus*), Temminck's red colobus (*Piliocolobus temminckii*), crowned sifaka (*Propithecus coronatus*), common squirrel monkey (*Saimiri sciureus*), large‐headed capuchin (*Sapajus apella macrocephalus*) and Gee's golden langur (*Trachypithecus geei*). Interestingly, there is no multi‐species genus or subfamily within which all species are largely pacific except for *Rhinopithecus* and possibly *Pygathrix*. While some species have an overall more pacific disposition towards other groups than other species, there is considerable intraspecific variation. For example, in bonobos pacifism in the context of between‐group interactions is more common in the Kokolopori and Wamba populations than in the LuiKotale and Lomako populations (Hohmann & Fruth, 2002; Fruth & Hohmann, 2018; Tokuyama, Sakamaki & Furuichi, 2019; Samuni, Langergraber & Surbeck, 2022).

It is possible that peace in primates is underreported because peaceful interactions are more easily missed compared to agonistic altercations. Also, because they are potent selective forces, conflict and competition have been prominent in the behavioural ecology literature, with a plethora of studies focusing on intergroup conflict. Lastly, intergroup interactions involving conflict are often classified as aggressive even though some individuals may have peaceful relations, thus inflating the true extent of aggression.

### (1) Intergroup tolerance and affiliation

Tolerant intergroup interactions are typically characterized by indifference and absence of agonism when two groups come across each other. Tolerance can be a dyadic or polyadic phenomenon. Robinson & Barker (2017, p. 2) define intergroup tolerance as “a state in which groups neither incur a net cost nor receive a net benefit as a result of interaction with other groups”. However, we argue that tolerance is not necessarily devoid of costs and benefits, and that, as long as the benefits exceed the costs for some individuals, tolerant intergroup interactions are liable to take place. Affiliative intergroup interactions require tolerance but also involve the exchange of socio‐positive behaviours between one or more individuals of different groups when groups are in proximity. Such behaviours seen include grooming, playing and food sharing (see Table 3). Such affiliations are often brief or occur as one‐off events, typically driven by immediate social needs (e.g. access to play partner).

**Table 3:** Behaviours observed during non‐agonistic encounters (see Fig. 2) between groups in non‐human primates. Species with multilevel societies are indicated by an asterisk.

Species	Behaviour	References
*Alouatta caraya*	Playing (mostly juvenile–juvenile); grooming and playing interactions (adult females, subadult males and females, juveniles, and infants)	Kowalewski (2007); Gennuso *et al*. (2018)
*Callithrix jacchus*	Non‐aggressive approaches, sexual behaviour	Lazaro‐Perea (2001)
*Callithrix penicillata*	Playing (infants), grooming, sexual contact	Decanini & Macedo (2008)
*Cebus capucinus*	“Dancing” [a form of relationship negotiation between males]	Perry (1996)
*Cercocebus galeritus*	Joint foraging, grooming, sexual presentation, mingling	Kinnaird (1992)
*Cercopithecus cephus*	Co‐sleeping, co‐moving, two groups in polyspecific association with two other guenon species	Gautier‐Hion *et al*. (1983)
*Cercopithecus mitis*	Playing by juveniles	Lawes & Henzi (1995)
*Cercopithecus neglectus*	Mingling	Gautier‐Hion & Gautier (1978)
*Chlorocebus pygerythryus*	Playing, mounting, grooming, touching	Cheney *et al*. (1981); Cheney (1981)
*Colobus angolensis palliatus*	Infant adoption, peaceful mingling, co‐resting, co‐feeding	Moreno‐Black (1974); Moreno‐Black & Bent (1982); Dunham & Opere (2016)
*Colobus angolensis ruwenzorii	Co‐travelling, co‐feeding, co‐resting, playing by infants	Stead & Teichroeb (2019); Miller *et al*. (2020); J. Teichroeb, pers. comm.
*Colobus angolensis cottoni*	Peaceful mingling, co‐travelling, co‐feeding	Bocian (1997)
*Eulemur rufus*	Sleeping or feeding in same or adjacent trees	Sussman (1972)
*Gorilla beringei*	Mingling, playing, touching	Sicotte (1993); Mirville (2018)
*Gorilla gorilla*	Co‐feeding, social play (between immatures but also between adults), mingling, nesting together overnight in close proximity	Parnell (2002); Bermejo (2004); Magliocca & Gautier‐Hion (2004); Forcina *et al*. (2019); Cooksey *et al*. (2020, 2025)
*Hylobates lar*	Co‐feeding, playing (infants, juveniles, subadults, adults), grooming, mating	Reichard & Sommer (1997); Bartlett (2003); Suwanvecho & Brockelman (2012)
*Hylobates funereus*	Co‐feeding, co‐travelling	Inoue *et al*. (2023)
*Lagothrix lagotricha*	Playing, grooming, copulating, mingling, co‐travelling, co‐feeding, co‐resting, peaceful visits to neighbouring groups	di Fiore (1997); Nishimura (2003); Di Fiore *et al*. (2009); Ellis & Di Fiore (2019)
*Lemur catta*	Co‐feeding, playing	Klopfer & Jolly (1970)
*Leontopithecus rosalia*	Food transfers	Troisi (2021)
*Macaca nemestrina*	Co‐feeding	Oi (1990)
*Macaca nigra*	Affiliation among males, males and females and among females (female affiliation included grooming and embraces)	Martinez Inigo (2018)
*Macaca sylvanus*	Mingling	Deag & Crook (1971); Deag (1973)
*Nasalis larvatus	In association; co‐travelling; sleeping in proximity	Bennett & Sebastian (1988); Yeager (1991); Boonratana (2002); Matsuda *et al*. (2024)
*Pan troglodytes*	Peaceful visits of parous females (with and without infants) and males (rare) to neighbouring communities, copulation, co‐feeding, associating, embracing, male–female and female–female grooming, exchanging greetings	Kawanaka (1982); Sugiyama (1999); Boesch & Boesch‐Achermann (2000); Williams *et al*. (2004); Boesch *et al*. (2008); Hashimoto *et al*. (2020)
*Pan paniscus*	Same‐sex (female–female & male–male) genital contact, copulations, playing, grooming; co‐feeding, co‐travelling, co‐resting, co‐nesting, food sharing, coalition formation, cooperative hunting, jointly mobbing predators, adoption	Idani (1990); Furuichi (2011); Fruth & Hohmann (2018); Sakamaki *et al*. (2018); Tokuyama *et al*. (2019); Furuichi (2020); Lucchesi *et al*. (2020); Samuni *et al*. (2020); Tokuyama *et al*. (2021); Cheng *et al*. (2022); Moscovice *et al*. (2022); Samuni & Surbeck (2023); Sakamaki & Tokuyama (2024); Samuni *et al*. (2024)
*Papio ursinus*	Mingling, sexual presentation, copulation, co‐feeding, co‐drinking, grooming, touching, mounting, embracing	DeVore & Hall (1965); Hamilton III *et al*. (1975); Cheney & Seyfarth (1977); Anderson (1981); Cowlishaw (1995); Kitchen *et al*. (2004)
*Papio cynocephalus*	Play bouts between juveniles, simultaneous use of a sleeping grove, co‐drinking, mingling at waterhole	DeVore & Hall (1965); Shopland (1982)
*Papio anubis*	Mingling of juvenile and subadult males, sexual presentation, co‐travelling	Packer (1979); Loftus *et al*. (2024)
*Papio papio	Male–male affiliation and coalitions, ritualized greetings, coordinated movements	Dal Pesco & Fischer (2018); Montanari *et al*. (2021); Dal Pesco *et al*. (2022)
*Papio hamadryas	Male–male coalitions, male–male affiliation (grooming), female–female grooming, coordinated movements	Stolba (1979); Abegglen (1984); Swedell (2002)
*Piliocolobus epieni*	Resting together during the night without mingling	Werre (2000)
*Piliocolobus tephrosceles*	Peaceful mingling, playing, copulation, female sexual presentation to male in other group	Clutton‐Brock (1972); Stanford (1998); Struhsaker (2010)
*Presbytis potenziani*	Playing, co‐feeding, co‐travelling, mingling	Fuentes (1994)
*Propithecus verreauxi*	Playing (including adults), non‐aggressive visits	Richard (1985); Brockman *et al*. (2001)
*Rhinopithecus bieti	Co‐travelling, sleeping and feeding in close proximity, temporary adoption of an infant, allonursing, juvenile/infant play groups, collective defence among males	Kirkpatrick *et al*. (1998); Ren *et al*. (2012); Grueter (2013); Zhu *et al*. (2016); Grueter *et al*. (2017a)
*Rhinopithecus roxellana	Co‐travelling, sleeping and feeding in close proximity, infant handling, unit mergers, females sexually presenting to male of other core units, extra‐unit copulation, collective defence among males, grooming with individuals in other units (one‐male units and all‐male units), juvenile play groups	Ren *et al*. (2000); Zhao *et al*. (2005); Zhang *et al*. (2008a); Zhang *et al*. (2006); Qi *et al*. (2014); Xiang *et al*. (2014); Wada *et al*. (2015); Qi *et al*. (2020); Xiang *et al*. (2022); Li *et al*. (2023)
*Rhinopithecus strykeri	Co‐travelling, sleeping and feeding in close proximity	Chen *et al*. (2015)
*Rhinopithecus brelichi	Co‐travelling, sleeping and feeding in close proximity	Bleisch *et al*. (1993); Nie *et al*. (2009)
*Rhinopithecus avunculus	Co‐travelling, sleeping and feeding in close proximity	Boonratana & Le (1998)
*Saguinus nigricollis*	Mingling, co‐travelling, joint hunting, co‐feeding, co‐resting	Izawa (1978); De La Torre *et al*. (1995)
*Saguinus fuscicollis*	Playing among young	Goldizen (1987)
*Saimiri sciureus*	Mingling	Terborgh (1983)
*Sapajus apella*	Co‐feeding	Defler (1982); Terborgh (1983)
*Sapajus nigritus*	Embracing, grooming, co‐resting	Scarry & Tujague (2012)
*Semnopithecus johnii*	Co‐feeding	Poirier (1968)
*Semnopithecus entellus*	Co‐feeding, co‐drinking from river, mingling	Yoshiba (1968); Hrdy (1977)
*Symphalangus syndactylus*	Co‐feeding	Palombit (1992)
*Theropithecus gelada	Play groups, affiliation (adult females and juveniles; rare), male–male coalitions, peaceful intermingling between two core units	Dunbar & Dunbar (1975); Mori (1979); Pappano *et al*. (2012); Gallo *et al*. (2021); Kifle (2024)
*Trachypithecus pileatus*	Mingling, sleeping in adjacent trees	Stanford (1991)

### (2) Intergroup coalitions

A more elaborate form of intergroup peace is intergroup coalitions, which often manifest as members of interacting groups engaging in joint action against a third party (conspecific or predator). The expression of such cooperative actions hinges on the existence of tolerant relationships between groups, with frequent tolerant interactions enabling cooperation to permeate group boundaries. There are a few non‐primate taxa in which such cooperative action has been documented. Territory‐owning Australian fiddler crabs (*Uca mjoebergi*) have been observed to help specific other crabs in defending their neighbouring territories against intruders (Backwell & Jennions, 2004). Cooperation between territorial neighbours has also been studied in some bird systems. Elfström (1997) reported that in rock pipits (*Anthus petrosus*) the arrival of an intruder resulted in an armistice between established, territorial neighbours and the coordinated eviction of the intruder. Similarly, in chipping sparrows (*Spizella passerine*) neighbouring rival males sometimes perform joint defensive responses when confronted with simulated territorial intrusions (Goodwin & Podos, 2014). In breeding pied flycatcher (*Ficedula hypoleuca*), individuals have been observed to leave their own territory and assist neighbours in the mobbing of a predator (Krams *et al*., 2008). Camerlenghi *et al*. (2023) demonstrated graded cooperative responses to distress calls that map onto the levels of the multilevel society of superb fairy‐wren (*Malurus cyaneus*).

**Fig. 4:**
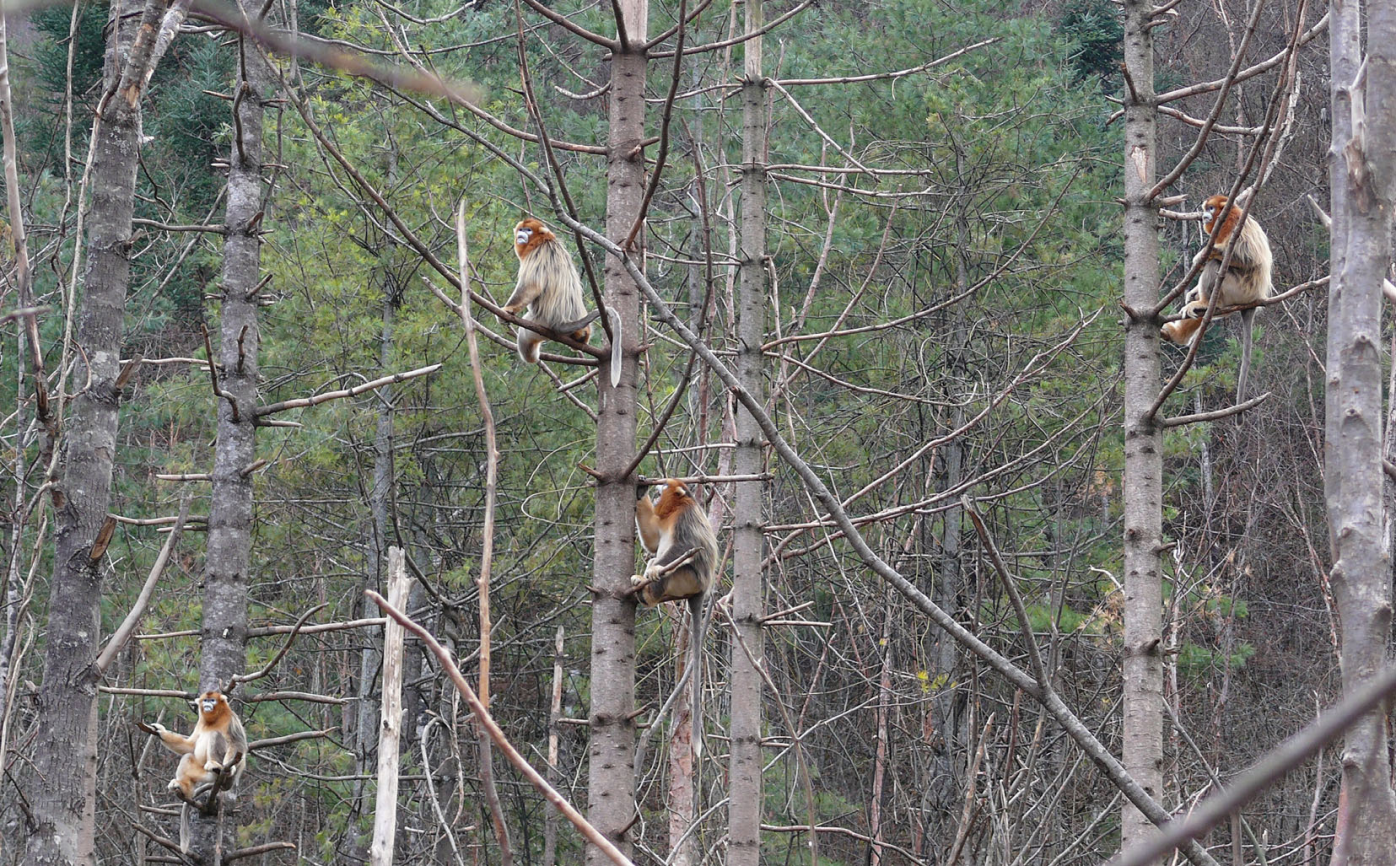
Golden snub‐nosed monkey (*Rhinopithecus roxellana*) males belonging to different core units sitting only meters apart in a tree and vigilantly scanning the surroundings for intruders. Photograph by Yang Yu.

A classic non‐primate mammalian example of between‐group cooperation is that of bottlenose dolphins in Shark Bay (Connor *et al*., 2022) where males form between‐group alliances, that is third‐order alliances. These are the outcome of preferential associations among second‐order alliances (a form of within‐group alliance) which support one another during contests over females (Connor *et al*., 2011; King *et al*., 2021). In African elephant (*Loxodonta africana*) multilevel societies, coalitions of higher‐tier units occasionally form in response to dominance interactions with outside elephants (Wittemyer, Douglas‐Hamilton & Getz, 2005). Such cooperation between social units appears to be uncommon in primates but has been observed in bonobos and in the multilevel systems of snub‐nosed monkeys (*Rhinopithecus* spp.), hamadryas baboons (*Papio hamadryas*), Guinea baboons (*Papio papio*) and geladas (*Theropithecus gelada*). Sakamaki *et al*. (2018) observed several instances where bonobo females of two separate groups joined forces in attacking a common target male during intergroup encounters. In golden snub‐nosed monkeys (*Rhinopithecus roxellana*) multiple “harem” males sometimes engage in joint patrolling and vigilance behaviour, which appears to be aimed at keeping satellite males in the bachelor group at bay (Krzton, 2011; Qi *et al*., 2014; Xiang *et al*., 2014; Huang *et al*., 2017) (Fig. 4). In geladas, it has been reported that the leader males of the core units sometimes simultaneously ward off bachelors (Wrangham, 1976) and may benefit from being close to preferred coalition partners during such events (Pappano *et al*., 2012). Iwamoto *et al*. (1996) describes a case of systematic mobbing of a leopard by several gelada males from different core units within a larger band. In multilevel Guinea baboons, males from different units within the same party (the second level of their multilevel society) form coalitions (Dal Pesco *et al*., 2022). In hamadryas baboons, observations of fights between adult males of different bands suggest that males within bands cooperate in protecting their females against foreign males, for example, by forming a common line of defence (Kummer, 1968, 1995; Sigg *et al*., 1982; Abegglen, 1984).

One unresolved question is whether coordinated collective action among members of separate social units (especially in the case of geladas and snub‐nosed monkeys) qualifies as genuine polyadic cooperation or whether the males resort to defensive action independently yet simultaneously when spotting an intruder. Simultaneous yet independent action can give an incorrect impression of cooperative behaviour. There is some anecdotal evidence for cooperation from gelada. Dunbar & Dunbar (1975) note that in situations where a gelada harem male is chased by males of the all‐male group, the harem male would often “yelp” and engage in display jumping and the throwing of objects. Wrangham (1976) interprets this behaviour as a form of advertisement that functions to alert other harem males to the presence of an all‐male group so as to elicit their help in chasing the bachelors away.

The mechanism underlying these cooperative acts also seems to vary among species. In dolphins and snub‐nosed monkeys, mutualism is the most likely candidate. In snub‐nosed monkeys, participation in defensive action against bachelors is mutually beneficial to all participating males because it reduces the risk of a takeover and reproductive losses. In pied flycatchers, the joint assault on a predator by breeding pairs fits either mutualism or tit‐for‐tat reciprocal altruism, depending on whether breeding pairs living in close or distant proximity are considered (Krams *et al*., 2008; Krama *et al*., 2012).

Vocal interactions between groups, while normally competitive in nature and aimed at managing spatial conflicts (e.g. Da Cunha & Byrne, 2006), may also be used for cooperative purposes. Anecdotal reports suggest that adult male Kloss's gibbons (*Hylobates klossii*) produce long‐distance alarm calls, which are audible far beyond the range needed to alert their immediate family. These calls may function to warn neighbouring groups, possibly related individuals, of potential danger (Tenaza & Tilson, 1977).

### (3) Visits

Another type of peaceful interaction spanning group boundaries is visits. These are reversible and thus different from dispersal which usually involves permanent relocation. Visits are not well documented in animals but there are some examples. Inter‐nest drifting by workers in social insects – whereby workers spend time in neighbouring non‐natal nests – can be construed as a form of visit (Hamilton, 1964; Lengronne *et al*., 2021). Few reports of visits exist in the primate literature. In common woolly monkeys (*Lagothrix lagotricha*), individuals sporadically leave their home group and enter other social groups where they remain for up to several days without eliciting any aggression from the residents (Nishimura, 2003; Ellis & Di Fiore, 2019). In Verreaux's sifakas (*Propithecus verreauxi*), individuals, especially subadult and younger adult males, sometimes pay a visit to neighbouring groups during the mating season. These visits are free of agonistic interactions and usually last from half a day to 2 days (Richard, Rakotomanga & Schwartz, 1993; Brockman *et al*., 2001). Peaceful intergroup visits of mothers and their infants lasting up to several days have also been observed in the Taï chimpanzees (*Pan troglodytes*) (Boesch *et al*., 2008). Even chimpanzee males, who are often portrayed as ultra xenophobic, have been observed to welcome and tolerate male visitors from other communities under certain conditions (Sugiyama, 1999), for example in small, isolated communities, where resident males may benefit from the recruitment of new males. In bonobos, young females may use tolerant inter‐community encounters as a platform for visiting neighbouring groups for short periods of time before finally settling in a new group (Sakamaki *et al*., 2015; see also Moscovice *et al*., 2022). Olive colobus (*Procolobus verus*) females often stayed in another group for a median of 1 day without immigrating. These visits are likely a strategy to expand the females' pool of mating partners (Korstjens & Schippers, 2003).

### (4) Mingling

Mingling between groups or multiple individuals from different groups is another example of intergroup peace. We use the term monospecific associations to refer to encounters between groups that are more than fleeting and that usually have a tolerant connotation (although occasional aggressive confrontations are inevitable when these associations are long‐lasting). While in association, these “supergroups” often perform activities in a coordinated and cohesive manner. Monospecific associations can be dichotomised into short‐term/temporary associations (lasting anywhere from a few hours to a few days) and long‐term/permanent associations (typically lasting years).

#### (a) Short‐term associations

In short‐term associations, social units intermittently encounter other social units upon which they mingle with them without any obvious signs of agonism. These associations typically last for a period of several hours (e.g. Tana River mangabey *Cercocebus galeritus*; Kinnaird, 1992). In the black‐mantled tamarin (*Saguinus nigricollis*), temporarily merged tamarin groups usually disbanded within half a day (Izawa, 1978). However, sometimes short‐term associations extend to several days. In bonobos, the maximum durations of bonobo between‐group encounters was 14 consecutive days (Samuni & Surbeck, 2023). Short‐term associations are characterized by some level of coordination between the conjoining groups. For example, the groups may move in parallel progressions (e.g. Kinnaird, 1992) or rest, travel, and forage together (e.g. di Fiore, 1997; Di Fiore *et al*., 2009). In Barbary macaques (*Macaca sylvanus*), when two groups united to form a larger conglomeration for several days, they moved and rested as one unit (Deag & Crook, 1971; but see Mehlman & Parkhill, 1988).

Short‐term associations usually involve two separate groups but can also involve multiple groups, for example 3–5 in tamarins (Izawa, 1978). An unusual form of mingling has been documented in woolly monkeys: here subsets of individuals from separate neighbouring social groups form subgroups that travel, feed, socialize and rest separately from the remaining members in each of their respective groups. These mixed‐group associations lasted from 1 h up to full days and sometimes persisted overnight (Ellis & Di Fiore, 2019). Mingling does not mean that group boundaries become completely blurred, for example in Tana River mangabeys each group remained largely discrete but sometimes there was mingling of individuals of the two groups (Kinnaird, 1992). In chacma baboons (*Papio ursinus*), however, there were instances where two associated groups became difficult to distinguish from a single large group due to extensive intermixing (Anderson, 1981). Similarly, DeVore & Hall (1965) mention gatherings of multiple baboon groups at two small, connected waterholes where the individuals were so close to one another that it was generally impossible to delineate the boundaries of the groups. Mingling may be accompanied by signals that facilitate group identification and contact maintenance such as long calls (De La Torre, Campos & De Vries, 1995).

**Fig. 5:**
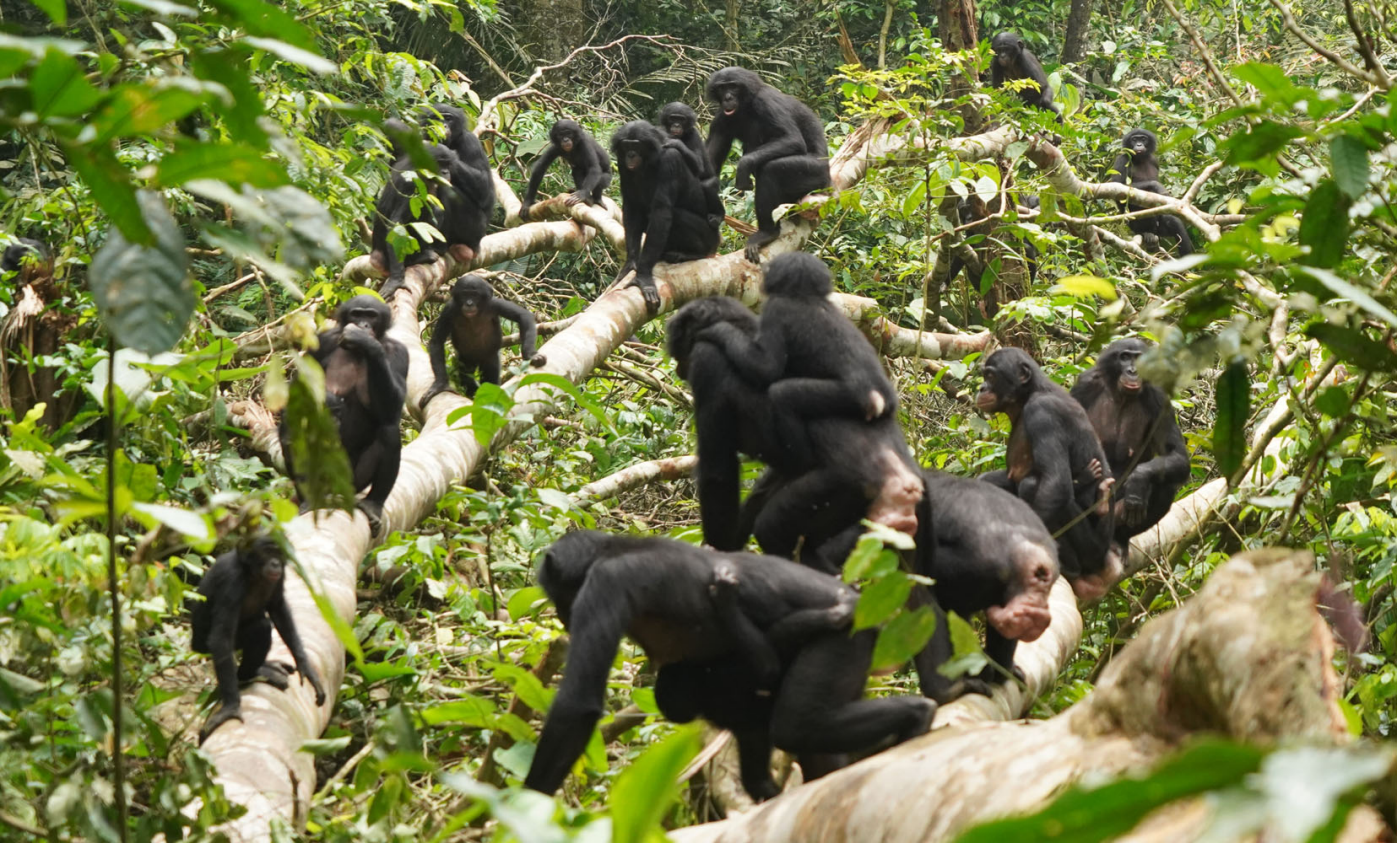
A peaceful encounter among bonobo (*Pan paniscus*) groups at Kokolopori, DRC. Credit: Liran Samuni/Kokolopori Bonobo Research Project.

Short‐term associations are particularly well documented in some ape taxa, that is bonobos, western gorillas, and gibbons. In bonobos, extended intergroup encounters or monospecific associations can include co‐utilization of feeding sites, resting and travelling together as well as the exchange of out‐group socio‐positive behaviours including sexual interactions, play, grooming, coalition formation and food sharing (Idani, 1990; Hohmann & Fruth, 2002; Furuichi, 2011; Fruth & Hohmann, 2018; Sakamaki *et al*., 2018; Moscovice *et al*., 2022; Samuni & Surbeck, 2023) (Fig. 5). Such encounters sometimes last for hours, days or even 2 weeks (Furuichi, 2020; Idani, 1990; Sakamaki *et al*., 2018). In Samuni & Surbeck's (2023) study, encounters averaged 12.5 h. Food sharing and grooming that occurs during associations often follows a reciprocal pattern, as indicated by a strong relationship between the grooming in‐degree (number of partners grooming the focal individual) and out‐degree (number of partner groomed by the focal individual) of bonobos between groups (Samuni & Surbeck, 2023). In some gibbon populations, intergroup interactions are sometimes peaceful and comprise mutual tolerance, co‐feeding and play (Reichard & Sommer, 1997) as well as prolonged affiliative associations between two groups (Fuentes, 2000). Western gorilla groups are often seen interacting non‐aggressively, feeding in close proximity, and playing with extra‐group members, sometimes for several hours (Forcina *et al*., 2019). They have also been observed sometimes to nest communally (Bermejo, 2004).

Some authors have proposed that due to the regular occurrence of pacific associations these species exhibit a higher‐level or community‐level organization within which groups are embedded (Fuentes, 2000; Bartlett, 2003; Reichard, Ganpanakngan & Barelli, 2012; Furuichi, 2019; Morrison *et al*., 2019). The term “community” can be misleading because this term is also used to describe chimpanzee and bonobo groups. Grueter & Wilson (2021) thus suggested use of the term “supra‐group organization” for species with regularly occurring mingling. Morrison *et al*. (2019) interpreted observations of western gorilla groups converging in forest clearings as evidence for the presence of a multilevel structure. However, given the transient nature of these encounters it may be more parsimonious to conclude that they also exhibit a supra‐group organization (Grueter & Wilson, 2021). Future research on the durability of these associations and the expression of partner preferences will illuminate these possibilities further (for a recent attempt, see Cooksey *et al*. 2025). There is also one non‐ape primate species – the Verreaux's sifaka – for which the existence of a supra‐group form of social organization (termed “neighbourhoods”) has been proposed (Jolly, 1966; Richard, 1985).

Polyspecific associations resemble short‐term monospecific associations in terms of their structural elements and the behaviours shown. Polyspecific associations are characterized by prolonged active proximity maintenance and activity coordination among two or more different, yet often phylogenetically closely related species (Rehg, 2016). Combinations of monospecific mingling and polyspecific associations have also been reported, for example two groups of moustached guenons (*Cercopithecus cephus*) in polyspecific associations with two other guenon species (Gautier‐Hion, Quris & Gautier, 1983). More transient and opportunistic encounters, typically in the context of co‐utilization of a resource patch, have been observed in many taxa. These do not qualify as true polyspecific associations (Waser, 1982; Stensland, Angerbjörn & Berggren, 2003). Irrespective of the type of mixed‐species associations (polyspecific associations *versus* more opportunistic encounters), xenophilic behaviours such as grooming, play, sexual interactions, food sharing, alloparental caretaking, and joint predator mobbing and territorial defence are occasionally observed between members of different species (Waser, 1980; Struhsaker, 1981; Buchanan‐Smith, 1990; Fimbel, 1992; Gathua, 2000; Pinheiro, Ferrari & Lopes, 2011; Eppley *et al*., 2015; de Carvalho Oliveira *et al*., 2017; Sanz *et al*., 2022; Falótico *et al*., 2021; Al‐Razi *et al*., 2023; Galotti *et al*., 2024). These can include species from distant phylogenetic branches within the order Primates [e.g. chimpanzees and guenons (Freymann *et al*., 2023); langurs and macaques (Nerlekar, 2012; Lee, Ang & Ruppert, 2021); langurs and gibbons (Wilcox *et al*., 2016); colobus monkeys and bonobos (Ihobe, 1990)] or even between members of different orders, for example macaques and deer (Vasava *et al*., 2013). Affiliation during polyspecific associations appears to be more common in species facing less interspecific competition, that is those with little or no dietary overlap (Struhsaker, 1981). Polyspecfic associations usually have been interpreted in terms of foraging advantages and antipredator benefits (Struhsaker, 1981; Terborgh, 1983; Cords, 1990; Stensland *et al*., 2003).

#### (b) Long‐term associations/multilevel societies

While the term “supra‐group social organization” applies to situations where sporadic and transient between‐group associations occur on a regular basis, the term “multilevel societies” describes situations where socio‐spatially distinct core units are integrated – in a pyramid‐like fashion – with other core units into lasting higher‐level social segments such as bands (Grueter *et al*., 2012a, 2012, 2017b, 2020). This societal arrangement is known to be present in some colobine monkeys [snub‐nosed monkeys (Kirkpatrick & Grueter, 2010); proboscis monkeys *Nasalis larvatus* (Matsuda *et al*., 2024); douc langurs *Pygathrix* spp. (Ulibarri & Gartland, 2021; Grueter *et al*., 2022); Adolf Friedrichs's Angolan colobus *Colobus angolensis ruwenzorii* (Stead & Teichroeb, 2019; Miller *et al*., 2020)], some papionins [hamadryas baboon (Kummer, 1968; Swedell & Plummer, 2012); gelada (Dunbar & Dunbar, 1975; Snyder‐Mackler, Beehner & Bergman, 2012); Guinea baboon (Fischer *et al*., 2017)], humans (Rodseth *et al*., 1991; Grueter *et al*., 2012a; Lehmann, Lee & Dunbar, 2014), some non‐primate mammals [e.g. African elephants (Wittemyer *et al*., 2005); plains zebra *Equus quagga* (Rubenstein & Hack, 2004)] and some birds (Papageorgiou *et al*., 2019; Papageorgiou & Farine, 2021; Grueter *et al*., 2021; Camerlenghi *et al*., 2022).

In multilevel societies, core units do not operate as individual social units but often synchronize their activities with other units (i.e. co‐feed, co‐sleep and co‐travel) and engage in collective decision‐making (e.g. Stolba, 1979; Montanari *et al*., 2021; Wu *et al*., 2023; for a non‐primate example see Maeda *et al*., 2021b). In some cases, sustained association and activity synchronization among units is all it takes for a multilevel society to materialize (Maeda *et al*., 2021b). However, the regular association among core units also creates opportunities for individuals to take part in friendly interactions (Table 3). These are most commonly seen among the lower levels, in particular between core units (and occasionally between a core unit member and a member of an all‐male unit; Li *et al*., 2023). While tolerance is required to stabilize the multilevel system, the close proximity among units and crowded conditions also make displacements and aggressive interactions (especially between males) unavoidable (e.g. Grueter *et al*., 2017a; Teichroeb *et al*., 2022). However, the establishment of a dominance hierarchy among units (Zhang *et al*., 2008b; Maeda *et al*., 2021a; but see Mori, 1979) may keep aggressive escalations in check. In some cases, tolerance is not only expressed among the constituent levels of a multilevel society but also between separate multilevel societies. For example, Bleisch & Xie (1998) found that bands of grey snub‐nosed monkeys (*Rhinopithecus brelichi*) did not show aggression when they encountered each other but rather fused together (although it is possible that there is a more inclusive level above the band in this species).

### (5) Communal roosting

The argument can be made that regular communal roosting is a form of intergroup peace. Coloniality is very common in some taxa [birds (Beauchamp, 1999); bats (Kerth, Wagner & König, 2001)] but uncommon in primates. In primates, communal roosting is seen predominantly in the form of shared use of cliff faces – in both multilevel societies (e.g. aggregations between bands in hamadryas baboons; Kummer, 1968) and non‐multilevel societies (olive baboons *Papio anubis*; Bidner, Matsumoto‐Oda & Isbell, 2018) – and riverine refuging in tree groves (proboscis monkey; Matsuda, Tuuga & Bernard, 2011). Communal roosting is largely the result of localized occurrence of suitable sleeping sites. We do not normally see any affiliation between social units while communally roosting but without some level of tolerance joint dormitory site use is not possible. Cliff use and riverine refuging are considered predation avoidance strategies, for example through dilution (Bidner *et al*., 2018).

### (6) Cross‐group adoption

Adoption of out‐group individuals represents an unusual variety of intergroup peace. There have been a few reports of this behaviour from different taxonomic lineages. Dunham & Opere (2016) describe a case where an adult female Angolan black‐and‐white colobus monkey (*Colobus angolensis palliates*) adopted an extra‐group infant alongside her own biological infant. Cäsar & Young (2008) noticed that an infant black‐fronted titi monkey (*Callicebus nigrifrons*), had moved from its natal group to another group where the adult female was breastfeeding it (alongside her own infant) and the adult male was carrying it. Tokuyama *et al*. (2021) report two cases of bonobo females adopting infants from different social groups and showing affectionate and nurturing behaviours such as carrying, grooming, nursing, and sharing food. There is also one case where cross‐group adoption was temporary and lasted only a few days: Ren *et al*. (2012) describe how an adult female snub‐nosed monkey from one core unit in a multilevel society nursed and cared for an infant from a different core unit of the same band. Lastly, there are two unique reports of cross‐genus adoptions: a melon‐headed whale (*Peponocephala electra*) calf by a bottlenose dolphin female (Carzon *et al*., 2019) and a common marmoset (*Callithrix jacchus*) infant by wild black‐striped capuchins (*Sapajus libidinosus*) (Izar *et al*., 2006). Explanations (ultimate and proximate) for the documented cases of within‐species, between‐group adoption include a strong female attraction to infants (Dunham & Opere, 2016; Tokuyama *et al*., 2021), high tolerance towards immatures and out‐group individuals (Tokuyama *et al*., 2021), poor infant recognition (Cäsar & Young, 2008), and “misdirected parental care” (Ren *et al*., 2012). The functional significance of cross‐group adoption remains obscure: indirect fitness benefits (kin selection) are less likely to be of significance in cross‐group adoptions than within‐group adoptions. A plausible functional explanation would be the accrual of direct benefits such as opportunities for practicing maternal skills (Riedman, 1982). However, the few observations of cross‐group adoptions have been explained with non‐adaptive explanations.

### (7) Mergers

Mergers refer to the partial or complete fusion of hitherto separate groups. These are on a different timescale than short‐term monospecific associations as these can last for many months or even be permanent. If social units lose their socio‐spatial distinctiveness upon amalgamation, mergers lead to a bigger mixed‐sex group, rather than a multilevel society. Mergers of two groups require high levels of intergroup peace and are a rather rare occurrence in mammals. Danaher‐Garcia *et al*. (2022) report a long‐term partial fusion of two communities of Atlantic spotted dolphins (*Stenella frontalis*) that lasted 9 months. One community moved into the range of another community which led to the formation of a mixed group. Some association levels between individuals from different communities were as high as the highest within‐community associations. A similar assimilation of two separate social clusters into a single community in this species was reported by Elliser, Volker & Herzing (2023). Baigger *et al*. (2013) describes the fusion of two social subunits (communities) of Bechstein's bats (*Myotis bechsteinii*), into a non‐modular colony in response to a strong population decline. In bonobos, there is a record of a fragmented group fusing with a neighbouring group (Hashimoto *et al*., 2008). For mountain gorillas, there are reports of male‐less groups of females and immatures merging with other groups (all‐male or mixed‐sex) upon the death of their silverback (Watts, 1989). In golden snub‐nosed monkeys, some one‐male, multi‐female core units in a multilevel society have been observed to merge with others, leading to the establishment of new social associations among individuals (Zhang, Watanabe & Li, 2008a). In these cases, one leader male remained in the merged unit and the other formerly resident male vanished. When subordinate units merged with dominant units, this resulted in the elevation of their rank (Zhang *et al*., 2008a). In some primates, mergers are the result of group size falling below a viable threshold (as a result of low birth rates and high mortality), as reported for vervet monkeys (*Chlorocebus pygerythrus*) (Isbell, 1990; Isbell, Cheney & Seyfarth, 1991) and Japanese macaques (*Macaca fuscata*) (Takahata *et al*., 1994; Sugiura, Agetsuma & Suzuki, 2002). In the case of Sugiura *et al*. (2002), the females of the adoptive group appeared to tolerate the unrelated immigrant females, whereas in the case of Takahata *et al*. (1994) affiliative social interactions between immigrant adult females and residents were rare. Mergers have also been achieved in captivity, as evidenced for example by the successful fusion of two zoo‐housed bonobo groups (Caselli *et al*., 2023).

## III. CHARACTERISTICS OF AFFILIATIVE INTERGROUP INTERACTIONS AND MINGLING

All non‐agonistic behaviours shown during intergroup encounters are also part of a species' within‐group repertoire. The benefits of these behaviours with group members (relationship formation, reproduction, practising skills for later use, etc.) may well extend to extra‐group members.

Grooming is the most common affiliative behaviour and can involve a variety of age–sex classes. Its role in hygiene and the establishment and maintenance of social bonds is well established (Dunbar, 1991; Grueter *et al*., 2013).

Play involving members from two groups is nearly exclusively found in juveniles/immatures (e.g. Fuentes, 1996; di Fiore, 1997). This behaviour may contribute to the development of social competence and behavioural flexibility (Spinka, Newberry & Bekoff, 2001; Palagi, 2018). When play involves individuals from other social units, it facilitates the development of an extended network that these individuals may rely on later in life (Mancini & Palagi, 2009). The occasional involvement of adults in intergroup play bouts has been recorded (Richard, 1985; Reichard & Sommer, 1997). Play appears to increase tolerance and reduce xenophobia between strangers (Antonacci, Norscia & Palagi, 2010).

Food sharing is an intricate expression of intergroup peace that has only recently been recorded in wild primate populations, most prominently in bonobos (Fruth & Hohmann, 2018; Moscovice *et al*., 2022); Moscovice *et al*. (2022) reported food sharing between females, while Fruth & Hohmann (2018) observed it between male–female and female–female participants. Troisi (2021) recently reported food transfer events between adult golden lion tamarins (*Leontopithecus rosalia*) belonging to different groups, but these were observed during an experiment. Other taxa have been recorded food sharing: certain ant species engage in non‐aggressive resource sharing with ants from other nests (polydomy) (Robinson, 2014), and in one cooperatively breeding species of bird (brown treecreeper, *Climacteris picumnus*), males were observed feeding nestlings, fledglings, and incubating females at nests in other territories within a supergroup assemblage (Doerr & Doerr, 2006).

Extra‐group copulations between members of distinct groups are widespread among mammals including primates (Lawler, 2007; Isvaran & Clutton‐Brock, 2007). These sexual acts generally appear to be “consensual” without the use of force and thus constitute an exchange of non‐aggression between members of two different groups. Extra‐group mating is a dyadic phenomenon that can occur during or outside an interaction involving entire groups. In the case of bonobos, between‐group interactions may be peaceful when matings take place, but matings can also occur when the overall nature of the encounter is agonistic. In fact, the intention to mate with out‐group members can be the reason for an overall agonistic encounter. For example, in banded mongoose, *Mungos mungo*, intergroup contests are the consequence of oestrous females seeking out males from neighbouring groups for mating (Cant, Otali & Mwanguhya, 2002; Nichols, Cant & Sanderson, 2015). This is an example of differences in individual priorities within groups. In black‐tufted marmosets (*Callithrix penicillata*), sexual contacts between members of different groups were often followed by agonistic behaviour from a third party against the intruder (Decanini & Macedo, 2008). In other species, such as bonobos, inter‐community associations are more likely to continue (less likely to be terminated) when extra‐community mating takes place between interacting individuals (Lucchesi *et al*., 2020).

Some intergroup encounters can have both agonistic and pacific elements, for example when periods of aggression are interspersed with episodes of mutual disregard and tolerant co‐feeding, as observed by Hrdy (1977) in Bengal sacred langurs (*Semnopithecus entellus*). Goldizen (1987) reported for brown‐mantled tamarins (*Saguinus fuscicollis*) that aggressive interactions are greatly reduced after the first hour or two of association and then play could take place between youngsters from the interacting groups. In bonobos, during the initial phases of intergroup encounters males may engage in displays and sometimes fights. However, aggression gradually reduces and females cross the perimeter of the other group and initiate grooming and genito‐genital rubbing bouts with extra‐group females (Furuichi, 2020). As stated above, extra‐group copulations can sometimes take place during agonistic intergroup encounters (Lazaro‐Perea, 2001; Cant *et al*., 2002; Nichols *et al*., 2015).

Age–sex classes often vary in their tendency to approach another group and initiate friendly interactions, with juveniles often more likely to do this than adults (Hamilton III, Buskirk & Buskirk, 1975; Cheney, Lee & Seyfarth, 1981). In black‐mantled tamarins, adult males and adult females tended to keep a distance from their counterparts in other groups when mingling, whereas subadults and juveniles mixed more freely with members of other groups: “Sub‐adults and juveniles moving in a temporarily‐merged group were mixed up, running about so busily that it was difficult to know from which group they came” (Izawa, 1978; pp. 262–263). In chimpanzees, males have an almost exclusively agonistic disposition towards males of neighbouring communities (Goodall, 1986; Wilson & Wrangham, 2003) whereas females in some populations occasionally exhibit non‐agonistic behaviours with male and female members of neighbouring communities (Kawanaka, 1982; Williams *et al*., 2004; Boesch *et al*., 2008; Hashimoto *et al*., 2020). Similarly, in bonobos, females from different groups are more likely to mingle and interact in the form of grooming or socio‐sexual behaviour whereas males of different groups tend to keep a distance from each other and interact less often (Idani, 1990; Furuichi, 2011). The more competitive approach of male bonobos to out‐group contact likely reflects protection of their own mating opportunities. However, in one population (LuiKotale), males and females are equally likely to perform affiliative out‐group social interactions (Moscovice *et al*., 2022). At Kokolopori, male bonobos actively groom out‐group males, hinting at the significant role such activities play in preserving peace among their neighbouring groups (Cheng *et al*., 2022). In Japanese macaques, males exhibited more affiliative behaviours towards extra‐group individuals than females, and subadult males were particularly likely to do so (Majolo, Ventura & Koyama, 2005). In line with standard socioecological reasoning, male participation in intergroup conflict reflects mate defence (or indirect mate defence through resource defence) whereas female participation is driven by the need to defend valuable resources (Wrangham, 1980; Fashing, 2001). Therefore, if between‐group mate competition is relaxed (e.g. outside the mating season; Cooper, Aureli & Singh, 2004), we may expect the sex whose reproductive success is more critically contingent on this form of competition (i.e. males; Trivers, 1972) to display an increased frequency of peaceful behaviour. Likewise, if resource competition is relaxed, we may expect females (whose reproductive success is more critically contingent on this mode of competition; Trivers, 1972) to show more peaceful behaviour.

Another variable that determines conduct during intergroup interactions is dominance rank. In vervet monkeys, low‐ranking females were more likely than those of high rank to initiate friendly interactions with females from neighbouring groups (Cheney *et al*., 1981). In Japanese macaques, low‐ranking males showed more affiliative behaviour towards foreign individuals than did high‐ranking males (Majolo *et al*., 2005).

## IV. FACILITATORS OF PACIFIC INTERGROUP INTERACTIONS

In this section we summarize various cognitive, socioecological and demographic factors that make it more likely that peaceful contacts will take place. Some of these facilitators or passive determinants are species specific (e.g. presence of male coalitions or shared decision making) whereas others vary contextually within species (e.g. group size and interaction location). Some of the facilitators discussed in this section are linked to certain hypotheses (e.g. pathogen stress hypothesis, habitat heterogeneity hypothesis, etc.), while others are more empirical in nature and have not been integrated within a theoretical framework (e.g. reproductive status of females).

### (1) Cognitive capacities

The idea that managing social relationships requires cognitive competence is encapsulated in the social brain hypothesis (Dunbar, 1998). This notion has traditionally been concerned with within‐group social challenges but has recently been extended to a between‐group context (Grueter, 2015; Ashton, Kennedy & Radford, 2020; H. Suter, C.C. Grueter, B. Ashton, I. Goncalves & A. Radford, in preparation). Peaceful between‐group contact inevitably leads to enlargement of an individual's social network, and to cope with the extra volume of social information generated, investments are needed in cognitive ability. The argument can also be made that managing peaceful interactions may be less cognitively taxing than managing agonistic ones. However, agonistic intergroup interactions may require group coherence for successfully mounting defensive strategies whereas peaceful interactions involve the cultivation of a larger relationship network. Species that exhibit social interactions both within and between groups may therefore be confronted with the challenge of handling concurrently relationships of very different types (i.e. strong and weak ties; Dunbar & Shultz, 2021). It thus is reasonable to predict that intricate peaceful contact between groups is more likely to evolve in species with larger brains. However, there is evidence from comparative analyses that species with more frequent intergroup encounters possess more elaborate secondary sexual traits (ornaments) that advertise competitive ability and genetic quality (Grueter & Lüpold, 2024). If these species rely on these ornaments to deduce relevant information about out‐group members, selection for individual recognition abilities may be attenuated.

### (2) Group size and composition

The relative competitive capacity or resource‐holding potential of opposing groups influences the likelihood of aggression during intergroup interactions. Game theoretic models predict that contest intensity increases when groups are more similar in size because assessing each other's relative competitive power is harder when groups are closely matched and this may lead to physical aggression to reach an outcome (Parker, 1974). Some studies support this prediction (e.g. Roth & Cords, 2016; Mirville *et al*., 2018b). However, in some species, contests are more likely to escalate when one group has a numerical/competitive advantage and uses its superiority to intimidate an opponent (Wilson *et al*., 2012). Variation in affiliation during intergroup interactions can also be influenced by the type of interactant. In mountain gorillas, males were less likely to engage in intergroup affiliation when the interactant was a solitary male (Mirville *et al*., 2018a).

### (3) Male coalitions

It is possible that species in which males are skilled at within‐group coalition formation are more likely to launch coalitionary attacks on neighbouring groups. It has been argued that the lack of strong male alliances may have reduced the propensity for escalated intergroup aggression in bonobos as compared to chimpanzees, fostering a more peaceful dynamic between groups (Ihobe, 1992; Wrangham, 1999). It is also possible that having access to male coalition partners within one's own group may eliminate the need to recruit defensive allies from other groups. This could explain why between‐group cooperation has been observed almost exclusively in monogamous birds and harem‐based multilevel societies.

### (4) Shared decision making

Hunt *et al*. (2024) recently analysed conflict decisions by groups using a modified version of the classic Hawk–Dove model. They found that animal species where movement decisions are democratic (i.e. shared across the group) are more likely to exhibit peaceful intergroup interactions than species where decisions are dictated by (belligerent) leaders. This argument is supported by the case of the vulturine guineafowl (*Acryllium vulturinum*) where democratic decision making and multilevel sociality co‐occur (Papageorgiou, Nyaguthii & Farine, 2024).

### (5) Population density

Population density may act as a mediator of intergoup conflict. For example, in Diana monkeys (*Cercopithecus diana*), aggression towards neighbours is contingent on local population density, with high population density (where intergroup competition is high) correlating with greater aggression and low density correlating with lower aggression (Decellieres, Zuberbühler & León, 2021).

### (6) Reproductive status of females

The reproductive state of females can also affect whether an intergroup interaction is peaceful or not. In chimpanzees, when encountering stranger females, males tended to interact peacefully with tumescent females but were more likely to attack non‐tumescent females (Williams *et al*., 2004). By choosing to mate with the presumably fertile females rather than exhibiting aggression, males enhance their potential for immediate reproductive success. Furthermore, there is a possibility that these females may integrate into the males' community. In chacma baboons, males actively try to prevent extra‐group males from accessing sexually receptive females. However, when oestrous females are absent, male displays tend to be less intense and shorter in duration, and peaceful interactions between groups are more likely to occur (Kitchen, Cheney & Seyfarth, 2004).

### (7) Interaction location

Encounter location has been shown to influence the competitive nature of an intergroup interaction. Groups tend to be more determined to defend aggressively areas of frequent use such as core areas containing valuable resources (Koch *et al*., 2016; Roth & Cords, 2016; Morrison *et al*., 2020).

### (8) Arboreality

Physical conflicts are generally riskier in trees (Wheeler, Scarry & Koenig, 2013) and are thus expected to occur less frequently above the ground.

### (9) Pathogens

According to the pathogen stress hypothesis developed for humans (Fincher & Thornhill, 2012), xenophobia is driven by fear or risk of infection with pathogens. Due to the elevated pathogen prevalence in warmer regions, stronger in‐group assortative sociality serves to mitigate infection risk by limiting contacts and interactions with outsiders and strangers. It is thus plausible that habitats with reduced diversity or prevalence of pathogens such as parasites (e.g. areas further away from the equator; Nunn *et al*., 2005) are more conducive to the establishment of intergroup peace.

### (10) Diet and resource distribution

Resources are intricately linked to the expression of tolerant or peaceful relationships between groups and can be a major disincentive for aggression. One precondition for intergroup peace is a lack or a limited degree of resource competition which relieves the constraints on aggregation. Grueter & van Schaik (2010) have shown that when increases in group size do not result in excessive energy expenditure because there is no need to increase foraging effort (low scramble competition), enduring associations between units (multilevel societies) are more likely to form. However, even when associating does carry energetic costs, such as longer daily path length in bonobos, these can be compensated for by visiting larger fruit patches (Lucchesi *et al*., 2021). Situations of low competition can ensue where resources are abundant or where resource defence is impractical, for example in a three‐dimensional habitat such as that inhabited by dolphins (Danaher‐Garcia *et al*., 2022). Patches of high‐quality clumped resources that are large enough to accommodate a group can be defended against other groups and are thus likely to elicit contest competition, as long as the costs of resource defence are not higher than the benefits of exclusive access (e.g. van Schaik, 1989; Grant, 1993; Sterck, Watts & van Schaik, 1997). Contrary to clumped/contestable foods, more evenly dispersed foods are generally more likely to result in peaceful intergroup interactions because they are less defensible. For example, in Tana River mangabeys, peaceful intergroup interactions generally occur when mangabeys eat uniformly distributed species and aggressive interactions are more common when they eat species with patchy distributions (Kinnaird, 1992). Female bonnet macaques (*Macaca radiata*) exhibit variation in aggressive tendencies depending on the pattern of local food distribution: individuals in a temple group with clumped food resources participated more aggressively in intergroup encounters than individuals in a forested habitat (Cooper *et al*., 2004).

Peaceful relationships can also emerge in situations of highly localized resources if these resource patches are of sufficient size to hold multiple units without causing friction. Geladas on alpine meadows are a good example of this scenario. DeVore & Hall (1965) describe joint usage of waterholes by multiple groups of baboons in Amboseli at the end of the dry season, with more than 400 individuals aggregating without disturbance.

The extent to which intergroup contact is pacific also covaries with seasonal or temporal fluctuations in resource availability. Peaceful interactions are expected to be more common during times of plenty such as peaks in the abundance of key foods. The benefits of aggressively displacing a neighbouring group are reduced when there is a generous supply of resources. Ulibarri (2013) showed for red‐shanked doucs (*Pygathrix nemaeus*) that greater young leaf availability (indicating relaxed ecological constraints) permits greater inter‐unit fusion through less competition. Recent studies on bonobos have shown that inter‐community tolerance can be facilitated by high fruit abundance or reduced food competition (Sakamaki *et al*., 2018; Lucchesi *et al*., 2020). However, one could also speculate that intergroup affiliation would be more common precisely when preferred resources are scarce, as such affiliation can foster tolerance around otherwise contested resource patches, and excessive energetic investments in defence are not viable during periods of scarcity when individuals are energetically stressed. Somewhat similar to this argument, there is evidence from territorial superb fairy‐wrens that they increase intergroup tolerance during the harsh resource‐scarce season (winter), thereby enabling individuals to exploit larger foraging areas (Camerlenghi *et al*., 2022, 2024).

Resource abundance and/or spatial concentration *per se* is not the only way tolerant intergroup relationships can form. Another key variable that may have far‐reaching consequences for the nature of between‐group relations is resource heterogeneity.

**Fig. 6:**
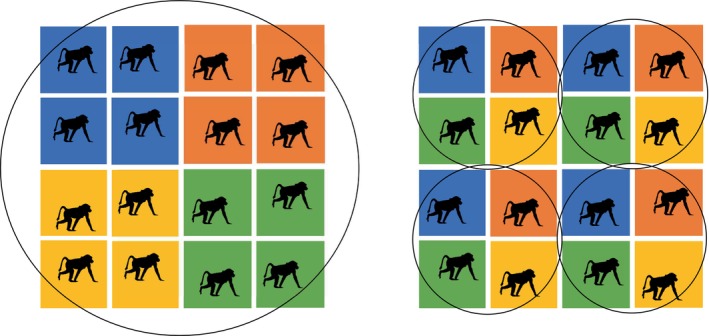
Hypothetical scenario depicting the conditions leading to intergroup tolerance in heterogeneous environments. Strict territoriality (black circles) is adaptive in a situation where resources are more evenly distributed and each of a set of neighbouring units has access to all essential habitat/resource types (right panel). However, where resources are patchy on a large scale, a group may end up being confined to a single habitat type and may not have access to critical resources in other habitat types and at other times of year. Here units are better off tolerating each other or even conglomerating into larger bands and sharing a large non‐territorial home range (large black circle; left panel). Each colour represents a different resource/habitat type.

The habitat heterogeneity hypothesis (Grueter, 2022; Grueter & White, 2014) posits that the need to access spatially and temporally heterogeneous resources may have relaxed intergroup relations. In strongly heterogeneous environments with patchy/dispersed resources, a primary group needs to cover a large range with sufficient exploitable resource patches to ensure year‐round access to all nutritionally important resources in all different habitat types. Other sympatric units of the same species will have the same spatial and dietary requirements and this ecological interdependence will inevitably result in overlapping home ranges and closer proximity of individuals from different groups (Fig. 6). Species with higher home range overlap show less aggression because maintaining exclusive access to the entire habitat mosaic by one unit would be unfeasible. This may render tolerance the more economic option in between‐group contexts. The habitat heterogeneity hypothesis is reminiscent of the resource dispersion hypothesis which was initially developed to explain group formation for solitary foragers (Carr & Macdonald, 1986; Johnson *et al*., 2002) but has not been explicitly applied to the formation of intergroup associations [although Macdonald & Johnson (2015, p. 99) do mention a “resource dispersion hypothesis community (a group of groups)”].

The habitat heterogeneity hypothesis makes two main predictions: (*i*) that greater resource heterogeneity leads to larger home ranges and greater home range overlap, and (*ii*) that greater home range overlap facilitates intergroup tolerance. Some support for the first prediction comes from Waser & Wiley (1979) whose comparison of five primate species in Kibale Forest showed that increasing variation in resource availability was correlated with a higher degree of spatial overlap of group activity range. In a preliminary test of the habitat heterogeneity hypothesis using a sample of Asian colobine species (which often prefer young foliage), there was a tendency for seasonal heterogeneity of foliage availability to be positively associated with home range overlap (Grueter & White, 2014). A recent study (Zhao *et al*., 2024) used a sample of 52 colobine species in a phylogenetic path analysis and found that climate heterogeneity (not resource heterogeneity) led to home range expansion, which then allowed for group size increases; while these authors did not investigate intergroup tolerance *per se*, group size can be used as a crude proxy for multilevel sociality and higher between‐group tolerance. Support for the second prediction comes from a comparative cross‐primate study showing that larger range overlap (more shared space among neighbours) is associated with fewer agonistic interactions (Grueter & Lüpold, 2024).

Heterogeneity and abundance may also interact and both may need to be present for more active forms of intergroup peace (i.e. cooperation) to emerge. Rodrigues, Barker & Robinson (2023) present a model showing that an environment where resources are both stable and relatively abundant is conducive to the establishment of tolerance between groups but not necessarily active cooperation between those groups. The authors suggest that the transition to active cooperation can be triggered when there is spatial or temporal heterogeneity within an environment of relative abundance.

Lastly, whether resource unpredictability has any bearing on intergroup peace in primates is uncertain. Temperate forests tend to be more predictable than tropical forests in terms of phenological patterns (Hanya, Tsuji & Grueter, 2013), and intergroup tolerance seems to be more common in temperate forests. This raises the possibility that spatial heterogeneity overrides the effect of temporal heterogeneity as a facilitator of intergroup tolerance.

### (11) By‐product of within‐group affiliation

It is possible that a pacific disposition in intragroup contexts is extrapolated to intergroup contexts. A comparative analysis across a sample of primate species showed that species devoting more time to within‐group grooming are less likely to be agonistic in encounters with out‐groups (C.C. Grueter & S. Lüpold, in preparation). The lower percentage of agonistic intergroup interactions in these species may thus be a by‐product of their more affiliative nature. This by‐product explanation may also apply at the individual level. In bonobos, individuals that showed higher within‐group food‐sharing or coalitionary tendencies were also more likely to form the same kind of connections with out‐group individuals (Samuni & Surbeck, 2023).

## V. DRIVERS OF PACIFIC INTERGROUP INTERACTIONS

For intergroup associations to become established as part of a species' behavioural repertoire the benefits of associating need to outweigh the costs. While the costs of aggressive interactions are well documented, costs can also arise during peaceful ones (e.g. diminished food intake when in the company of many others, risk of infanticide from unrelated males). In this section we discuss the drivers of pacific interactions among individuals from different groups. The adaptive fitness benefits that individuals gain from peacefully interacting with out‐group individuals include dispersal facilitation, information gathering (e.g. reconnaissance before transfer), reduction of predation risk, facilitation of resource exploitation, reciprocal resource access, joint resource and mate defence, and extra‐group mating. Peaceful intergroup encounters offer a chance for social network expansion, with its concomitant benefits (and costs). However, limited empirical data exist for assessment of the ultimate conditions enabling these factors to promote the peaceful coexistence of groups. It can be difficult to determine whether these factors promoted the evolution of tolerance or whether they became relevant only once tolerance had been established. For example, dispersal, information exchange and extra‐group mating will be easier when some level of tolerance exists. Hence, distinguishing between drivers and stabilizers can be challenging.

### (1) Dispersal facilitation

Friendly intergroup contacts with their associated socio‐positive behaviours can provide individuals with opportunities for transfer to other groups (Idani, 1990; Nishimura, 2003; Moscovice *et al*., 2022). However, while peace facilitates intergroup transfers, it is not mandatory. Transfers can also occur when groups are engaged in hostile interactions (e.g. Sicotte, 1993).

### (2) Information gathering

The role of peaceful interactions with other groups in facilitating pre‐dispersal prospecting has been noted by several primatologists (Lazaro‐Perea, 2001; Sicotte & Macintosh, 2004; Di Fiore *et al*., 2009; Hashimoto *et al*., 2020). In bonobos, young females may take advantage of intergroup associations to identify a suitable group for future transfer and reproduction (Sakamaki *et al*., 2015; Sakamaki & Tokuyama, 2024). Perry (1996) considered it likely that male white‐faced capuchins (*Cebus capucinus*), use non‐aggressive intergroup encounters to become familiar with males in other groups and assess their options for transfer in the future. Assessment of breeding opportunities has also been identified as an important function of non‐aggressive intergroup contacts in birds (Hale *et al*., 2003). The ability to obtain information about mating opportunities in other groups is not contingent on peaceful interaction; some individuals may be engaged in displays or disputes while others are mate prospecting. Peaceful encounters not only allow individuals to assess the suitability and quality of potential mates, but also allow evaluation of the competitive ability of same‐sex competitors (although this may be assessed more easily during aggressive encounters), which may assist in planning a takeover (Sicotte & Macintosh, 2004).

### (3) Protection from predators

Maintaining proximity to or clustering together with neighbouring units may reduce predation threat through shared vigilance or dilution effects. This has been shown for polyspecific associations (e.g. Wolters & Zuberbühler, 2003) but may also explain tolerance between conspecifics from different groups (Adams *et al*., 2021; Decellieres *et al*., 2021). For example, multilevel grouping in female sperm whales (*Physeter macrocephalus*) in the Pacific has been attributed to protection against predation by killer whales (Whitehead *et al*., 2012). In African elephants, associations at higher levels may emerge in reaction to pressures exerted by predators (Wittemyer *et al*., 2005). Having access to a pool of individuals with whom they can engage in communal predator deterrence is an incentive for members of a unit to remain in proximity to other units (Iwamoto *et al*., 1996).

### (4) Facilitation of resource acquisition

Boonratana (2002) suggested that banding in proboscis monkeys might enable them to exploit large food sources that are very unpredictable in space and time. Kirkpatrick *et al*. (1998) used a hypothesis originally proposed by Cody (1971) and Altmann (1974) to argue that band formation in black‐and‐white snub‐nosed monkeys (*Rhinopithecus bieti*) could enhance foraging returns by reducing the risk of encountering already depleted patches (but see Grueter & van Schaik, 2010). Research on bonobos has revealed that the probability that an intercommunity association would end was lower when the benefit from the association was more efficient foraging in less familiar areas (Lucchesi *et al*., 2020). Improved foraging efficiency is also a driver of polyspecific associations (e.g. Terborgh, 1983).

### (5) Reciprocal resource access

When there is high variance in resource access among groups, animals will gain from reciprocal access to these resources (Kelly, 2013; Jaeggi *et al*., 2016). In the facultatively polydomous wood ant *Formica lugubris*, resources are exchanged directly from nests with a foraging surplus to nests with a foraging deficit (Ellis, Franks & Robinson, 2014). The existence of reciprocal resource access in primates is speculative and requires further long‐term data. Drawing parallels with human gatherer–hunters where investments in intergroup relationships provide benefits in terms of shared access to neighbouring group territories (Cashdan, 1983), Morrison *et al*. (2020) proposed that the higher levels of affiliative behaviour they found between mountain gorilla groups after group fission could enable reciprocal range access with a reduced risk of aggression. Moscovice *et al*. (2022) could not test directly for reciprocal resource buffering in their bonobo study population but they recorded three lines of evidence that support this scenario: incursions into a neighbouring group's range led to access to higher‐quality food for the focal group, most intergroup encounters were of extended duration and involved affiliative exchanges, and tolerant incursions also occurred in the reverse direction. Robinson & Barker (2017) argue that a possible reason for the rarity of reciprocal resource access in primates is that it requires delayed reciprocity, which is susceptible to cheating.

### (6) Joint resource defence

A benefit of intergroup peaceful associations, which has been advanced to explain group mergers, is that they confer an advantage in intergroup conflicts over resources. This is a reasonable conclusion in light of the observation that group mergers are often preceded by decreases in group size below a sustainable threshold (Hauser, Cheney & Seyfarth, 1986; Isbell *et al*., 1991; Takahata *et al*., 1994). It has also been proposed that the maintenance of mixed‐species associations in tamarins is driven by the benefit of enhanced resource defence during intergroup encounters (Garber, 1988).

### (7) Collective/communal mate defence

Communal defence is usually aimed at defending “reproductive capital” from competitors and thus by maximizing lifetime fitness. By keeping competitors such as bachelor males away through cross‐group collective action, the risk of a group takeover and subsequent infanticide can be minimized (Wrangham, 1976; Rubenstein & Hack, 2004; Grueter & van Schaik, 2010; Xiang *et al*., 2014). Coalitionary attacks by females on a target male, as observed in bonobos, can also serve to reduce male harassment (Tokuyama *et al*., 2019). Successful coalitionary acts require investment in relationship building among females in different groups.

### (8) Extra‐group mating

Extra‐group copulations can confer both direct and indirect benefits to females. For example, polyandrous mating, *via* paternity confusion, can reduce the probability of infanticide (Lukas & Huchard, 2014). Qi *et al*. (2020) provided evidence that in multilevel societies of golden snub‐nosed monkeys, females mate with males outside their core unit to lower the risk of infanticide if one of those males subsequently establishes himself as the leader of their core unit. Another reason for females to seek out extra‐group matings is to obtain “good genes” or heterozygosity‐enhancing genes for their offspring (Foerster *et al*., 2003). Extra‐group mating can thus be considered an alternative to dispersal to reduce the risk of inbreeding within groups. Extra‐group mating can be more common when natal dispersal is limited (i.e. when breeding with in‐group members would increase inbreeding depression; e.g. Pilot, Dahlheim & Hoelzel, 2010). Males also benefit in terms of reproductive success (e.g. Lawler, 2007). Despite the potentially positive fitness implications of mating with out‐group females, males may also suffer fitness losses by being unable to prevent their own females from mating with out‐group males (Tokuyama *et al*., 2019).

## VI. STABILIZERS OF PACIFIC INTERGROUP INTERACTIONS

### (1) Relatedness and familiarity

While the drivers discussed above may be sufficient for tolerant between‐group associations to emerge, a further mediator of xenophobia and intergroup agonism is the degree of relatedness and familiarity between interacting groups. Genetic differentiation between groups can be reduced when individuals mate with members of other groups. Successful extra‐group matings thus can result in an extended kinship network that may strengthen intergroup associations. Rudimentary forms of intergroup integration can also result from non‐random dispersal. Relations between groups that have exchanged individuals in the past should be more peaceful than relations between groups that have not, since individuals in the former groups are likely to be related to each other (Alexander, 1975; Cheney, 1981).

Familiarity, the other major stabilizer, will increase when groups meet one another regularly and/or when individuals have a common history with some individuals in other groups (e.g. shared group membership prior to dispersal or group fission). Tolerant relations can then develop through this familiarity. When familiarity and consequent conflict reduction arise from sharing a common territorial border, this is known as the “dear enemy” scenario (Getty, 1987; Christensen & Radford, 2018). For example, great tits (*Parus major*), are more likely to participate in a neighbour's nest defence if they shared a territory boundary with that neighbour the previous year (Grabowska‐Zhang, Sheldon & Hinde, 2012). Familiarity can influence tolerance separately from relatedness but often these two variables work in conjunction and are difficult to disentangle.

There is good evidence from a variety of taxa that kin selection may be an organizing principle at the between‐group level. In African elephants, relatedness between older females predicted temporary fusion between core social groups (Archie, Moss & Alberts, 2006). In Przewalski's horses (*Equus ferus przewalskii*), harems of genetically related stallions were closer to each other in the network, and female exchange was more frequent between closer harems (Ozogány *et al*., 2023). In geladas, female–female relatedness significantly predicted associations between core units (Snyder‐Mackler, Alberts & Bergman, 2014). Incidents of peaceful interactions between mountain gorilla groups appear to be at least partially determined by kin relationships across group lines (Mirville *et al*., 2018b). In a population of western gorillas, peaceful coexistence among neighbouring groups may be mediated by a dispersed network of related males (Bradley *et al*., 2004). For gibbons, multiple factors may explain intergroup tolerance, including the existence of extra‐group copulations and short dispersal distances, which mean that neighbouring groups may contain close relatives (Reichard & Sommer, 1997; Bartlett, 2003; Matsudaira *et al*., 2018). This has led to the suggestion that gibbon groups reside within larger communities (Fuentes, 2000; Bartlett, 2003; Reichard *et al*., 2012) or exhibit a supra‐level organization (*sensu* Grueter & Wilson, 2021).

### (2) Relatedness and familiarity in the context of post‐dispersal associations

Tolerance between groups can be influenced by the recent history of transfers of either males or females between groups. In Verreaux's sifakas, intergroup transfer by males, coupled with regularly occurring intergroup encounters, visits, and extra‐group mating, links groups into “socioreproductively significant neighbourhoods” (Richard *et al*., 1993, p. 2). In Adolf Friedrichs's Angolan colobus, when males transferred between core units in their multilevel society, their former and new units exhibited significantly higher association indices with one another than with other core units for 1–2 months post‐dispersal. Here dispersal does not immediately sever ties to their former units (Adams *et al*., 2021). In bonobos, the transfer of females between groups influences the affiliative relationships when two groups meet subsequently, so females may be important mediators of peace between groups (Furuichi, 2020, 2011). In Temminck's red colobus, the reaction of a female toward an opposing troop was more tolerant if she had previously been a member of that group (Starin, 1991). In a golden snub‐nosed monkey band, female intraband dispersal had the effect of reducing the clustering coefficients, thus bringing the core units of their multilevel society closer together (Fang *et al*., 2022). In western gorillas, focal groups were more tolerant and affiliative with subadult males who had dispersed to neighbouring but spatially close areas (Cooksey *et al*., 2020). There are also examples where males and females react differently to encounters with familiar groups. Vervet monkey females were most aggressive to groups from which males had *not* transferred during the study period whereas males were most aggressive to groups from which one or more of them had previously transferred (Cheney, 1981).

Several authors have commented on how relatedness and familiarity affect post‐fissioning associations/tolerance. In tufted capuchin monkeys (*Cebus apella nigritus*), two groups that had fissioned a few years previously did not show direct physical aggression upon encountering one another, and instead often used affiliative contact (Scarry & Tujague, 2012). Hamilton III *et al*. (1975) reported that patterns of encounter in chacma baboons are conditioned by their historical relationships. Two groups that were apparently fissioning fragments of a larger troop coalesced peacefully when they met without showing confrontational behaviour. Similar examples of group fissioning leading to (at least initially) tolerant interactions between newly established groups have been reported in rhesus monkeys (*Macaca mulatta*). Missakian (1973) notes that, in the initial stages of new daughter‐group formation, adult females of the three groups continued to groom across group boundaries when the groups were adjacent to one another. In mountain gorillas, familiarity of interacting groups (i.e. whether they had split from a single group in the past) was the main determinant of peacefulness during intergroup encounters (Mirville *et al*., 2018, 2018) but not when they occurred within core areas (Morrison *et al*., 2020). However, familiarity fails to attenuate conflict in some cases. When a chimpanzee community breaks up, shared recent ancestry and long‐term familiarity does not prevent the descendent groups from becoming antagonistic towards each other (Goodall, 1986; Sandel & Watts, 2021). And newly fissioned groups of blue monkeys (*Cercopithecus mitis*) engage in intense aggressive interactions as they divide their original shared home range into two distinct territories (Cords & Rowell, 1986).

## VII. RESEARCH DIRECTIONS

Some of the preconditions for intergroup peace require more research, for example what cognitive capacities are necessary for forming affiliative relationships and maintaining them after groups separate? How do these relationships differ between humans and non‐humans? We also need a more refined understanding of the link between intergroup peace and the characteristics (distribution and abundance) of essential resources. Systematic cross‐species tests like those used by Zhao *et al*. (2024) are needed to substantiate the association between resource heterogeneity (in both space and time) and intergroup tolerance. In the case of monospecific associations, it will be of interest to consider the extent to which these are driven by social attraction or shared preferences for resource hotspots. While intergroup peace is more common in certain species than others, there is also intraspecific variation in intergroup relationships and social strategies which is likely mediated by socio‐ecological factors.

There are also potential morphological and genetic correlates of intergroup peace. In humans, skeletal and cranial gracility can be used as an indicator of reduced reactive aggression (Wrangham, 2019), enabling out‐group tolerance and affiliation with unfamiliar individuals. Is there any evidence for covariation between morphological gracility (e.g. in bonobos) and intergroup tolerance in non‐human primates? Schahbasi, Huber & Fieder (2021) argued that, if ethnic nepotism results in high levels of inbreeding, individuals may be programmed to seek increased out‐group contact. Are genetically more inbred groups more open to strangers? Or does extra‐group mating effectively prevent high levels of inbreeding in undisturbed populations (e.g. Pilot *et al*., 2010)?

Pacific intergroup interactions are underreported in the literature with few detailed descriptions. We thus need more informative accounts on how exactly such interactions unfold, for example who/what enables xenophobia to be overcome (e.g. play; Antonacci *et al*., 2010). Various behaviours have been implicated in facilitating intergroup peace, but there may also be more subtle cues involved, for example visual monitoring. Contact/loud calls may also serve as a tool to facilitate friendly encounters and maintain between‐unit cohesion. Loftus *et al*. (2024) recently examined how nighttime associations and social dynamics between groups influence daytime associations. Following the communal use of a sleeping site, neighbouring baboon groups showed a marked inclination for prolonged interactions, illustrating deliberate and non‐random affiliations between the groups. Similar data could be collected for other species with tolerant intergroup relationships. The formation of intergroup coalitions is a particularly complex expression of intergroup peace, but little is known about how they are mechanistically sustained, for example through reciprocity or mutualism.

The endocrinological machinery controlling between‐group interactions in primates has been explored in the context of intergroup conflict (Samuni *et al*., 2019; Triki, Daughters & De Dreu, 2022), but not intergroup affiliation (for humans, see Marsh *et al*., 2017). Hormonal data could also clarify if peace is “genuine” or simply reflects the successful suppression of aggressive tendencies.

One tantalizing question pertains to the role of personality of certain group members in instigating and participating in socio‐positive intergroup interactions. Mirville (2018) found suggestive evidence that male mountain gorillas that were more “open” were more likely to engage in cross‐group affiliative exchanges. There are also intriguing reports of a Japanese macaque population exhibiting higher‐than‐average levels of intergroup peace which may attributable to a greater concentration of genetically mediated tolerant personality types in that population (Zhang & Watanabe, 2012; see also Inoue‐Murayama *et al*., 2010). It may be the case instead that the propensity to engage affiliatively with out‐group members is a socially learned trait, through exposure to this behaviour. Another question of interest is whether a tolerant disposition in a within‐group context extends to a between‐group context (see Section IV.11).

When tolerant interactions with neighbours occur on a regular basis, they can reshape the fitness landscape of their interactants. Fitness consequences can be measured through reproductive success or surrogates such as energy balance or changes to telomere length. Intergroup contact also provides an arena for the spread of cultural variants, although it does not appear that species with higher levels of intergroup tolerance and mingling are the most culturally sophisticated, but this requires empirical validation. We also need a better understanding of the epidemiological consequences of peaceful intergroup associations as expanding one's circle of interaction may provide a route for transmission of parasite and diseases (Craft *et al*., 2011; Ryu *et al*., 2020). The increased social contact resulting from peaceful encounters may expose individuals to a larger pool or higher diversity of gut microbiota with its protective effects such as improved disease resistance or boosted immune response (Amato, 2016).

When more data on pacific interactions in a wider range of primate taxa become available, formal phylogenetically controlled comparative analyses such as phylogenetic generalized least squares regressions or phylogenetic path analyses would allow us to examine various socioecological and environmental correlates and drivers of intergroup peace. There also exists significant potential for the advancement of theoretical modelling of xenophilic conditions (e.g. Rodrigues *et al*., 2023), similar to modelling of multiparty contests with non‐group members (Sherratt & Mesterton‐Gibbons, 2013). Given the difficulties of measuring ecological variation in the field, agent‐based models may be particularly suitable for exploring how pacific interactions emerge in response to variation in socioecological contexts and resource characteristics (Santos *et al*., 2015; Spikins *et al*., 2021). Virtual world experiments (e.g. Wilson *et al*., 2020) could be used to model the effects of resources on interactions with out‐group avatars.

## VIII. CONCLUSIONS

(1) Peaceful interactions and relationships in primates and other animals extend beyond the confines of the in‐group, and can be an essential and adaptive part of intergroup relationships. We distinguished among multiple forms of xenophilia, that is intergroup tolerance, intergroup affiliation, visits, mingling, intergroup cooperation/coalitions and mergers between groups.
(2) Intergroup peace can be investigated by dissecting it into facilitators, drivers, and stabilizers. Its degree of expression in a species is expected to be contingent on which of these forces are at play. One important facilitator is resource distribution. Resource heterogeneity can lead to home range overlap among groups which then facilitates tolerance and intergroup associations. Key drivers of intergroup peace include dispersal facilitation, information gathering, predation protection, extra‐group mating, communal defence, and reciprocal resource access. Important stabilizers are the patterns of relatedness and familiarity among individuals in different groups. These stabilizing mechanisms can make groups become less distinct.
(3) The benefits of peaceful interactions can accrue to individuals, subsets of individuals, or entire groups. When individual interests align, these benefits can extend to all group members. However, when optimal strategies among individuals differ, the distribution of benefits may become uneven, leading to potential conflicts.
(4) We call for field primatologists to pay more attention to hitherto rather neglected forms of between‐group contact that do not involve aggression. We hope that this review will stimulate empirical and theoretical research in this field. It is possible that expressions of intergroup peace will be impacted by global change (climate change or anthropogenic activities) increasing the probability of conflict and competition between populations.
(5) An understanding of the functional correlates of peaceful between‐group relationships holds promise for extrapolation to human social systems. One of the defining features of human multilevel sociality is our ability to overcome hostility and form tolerant and cooperative relationships with other groups (Fry, 2006; Grueter & White, 2014; Hames, 2019; Pisor & Surbeck, 2019; Glowacki, 2024). Intergroup peace may not be a human derived trait as various forms of it have evolved on multiple phylogenetic branches. However, the extent of peaceful coexistence and interactions may lie at the heart of human success as it is a fundamental prerequisite for cultural knowledge to pass beyond a single group and accumulate in complexity through population connectivity and demographic reinforcement (Powell, Shennan & Thomas, 2009; Spikins *et al*., 2021). Comprehending the evolutionary roots of tolerance and cooperation will remain a top priority if we are to address global problems such as climate change, poverty, famine, war, and disease.

## Supporting information


**Table S1.** Percentage of intergroup encounters that are completely or largely devoid of aggression. These are divided into (1) neutral/tolerant/ignore and (2) affiliative/peaceful/mingling. Where multiple studies/populations contributed to the data set we calculated the average percentage. The qualitative frequency descriptors (e.g. rare, common) are taken from the original sources and cannot be reliably linked to specific numerical values. Species with multilevel societies are indicated by an asterisk.
