# Supplementary material for: Close encounters of the friendly kind: pacific between‐group interactions in primates
Source: Biol Rev Camb Philos Soc. 2025 Jun 16;100(6):2257–84. doi: 10.1111/brv.70046 (PMC12586289; doi:10.1111/brv.70046)
Supplement: Supplementary file 1 — Table S1. Percentage of intergroup encounters that are completely or largely devoid of aggression. These are divided into (1) neutral/tolerant/ignore and (2) affiliative/peaceful/mingling. Where multiple studies/populations contributed to the data set we calculated the average percentage. The qualitative frequency descriptors (e.g. rare, common) are taken from the original sources and cannot be reliably linked to specific numerical values. Species with multilevel societies are indicated by an asterisk. [file BRV-100-2257-s001.docx]

**Table S1.** Percentage of intergroup encounters that are completely or largely devoid of aggression. These are divided into (1) neutral/tolerant/ignore and (2) affiliative/peaceful/mingling. Where multiple studies/populations contributed to the data set we calculated the average percentage. The qualitative frequency descriptors (e.g. rare, common) are taken from the original sources and cannot be reliably linked to specific numerical values. Species with multilevel societies are indicated by an asterisk.

| **Species** | **% neutral/**  **tolerant/ignore** | **% affiliative/**  **peaceful/mingling** | **Sources** | **Notes** |
| --- | --- | --- | --- | --- |
| *Alouatta belzebul* | 52.4 |  | Pinto *et al*. (2003) |  |
| *Alouatta caraya* | yes | 41 | Kowalewski (2007) | Group members engaged in affiliative interactions during 41% of intergroup encounters → does not mean these were exclusively affiliative |
| *Callithrix penicillata* |  | rare | Decanini & Macedo (2008) |  |
| *Callithrix jacchus* |  | 7.4 | Lazaro-Perea (2001) | 7.4% were exclusively affiliative but affiliation was observed in 48% of encounters |
| *Cercocebus galeritus* |  | 6.4 | Kinnaird (1992) |  |
| *Cercopithecus ascanius* | 51 |  | Brown (2013); M. Brown, unpublished data |  |
| *Cercopithecus mitis* | 57 | yes | Lawes & Henzi (1995) |  |
| *Cercopithecus neglectus* |  | yes | Gautier-Hion & Gautier (1978) |  |
| *Chlorocebus pygerythryus* |  | 20 | Cheney (1981, 1987) |  |
| **Colobus angolensis ruwenzorii* | mostly |  | Bocian (1997); Miller *et al*. (2020); Stead & Teichroeb (2018) |  |
| *Colobus polykomos* |  | 12 | Korstjens *et al*. (2005); Korstjens (2001) |  |
| *Colobus vellerosus* | 12.7 |  | Teichroeb & Sicotte (2018) |  |
| *Eulemur fulvus* | yes |  | Sato (2013) |  |
| *Gorilla beringei* |  | 12.5 | Mirville *et al*. (2018*b*); Sicotte (1993) |  |
| *Gorilla gorilla* | 61.2 | 10.5 | Bermejo (2004); Caillaud *et al*. (2008); Cooksey *et al*. (2020); Doran‐Sheehy *et al*. (2004); Forcina *et al*. (2019); Greenway (2015); Magliocca & Gautier-Hion (2004); Parnell (2002) | Minimum estimate; probably higher but Forcina *et al*. (2019) (who report that all intergroup interactions were non-aggressive) did not provide % neutral *versus* % affiliative |
| *Hapalemur griseus alaotrensis* | 44 |  | Nievergelt *et al*. (1998) |  |
| **Homo sapiens* | yes | yes |  |  |
| *Hylobates klossi* |  | yes | Tenaza (1975) |  |
| *Hylobates lar* | 6 | 17 | Bartlett (2003) |  |
| *Hylobates pileatus* |  | 41 | Suwanvecho & Brockelman (2012) |  |
| *Indri indri* | yes |  | Powzyk (1997) |  |
| *Lagothrix lagotricha* | 60 | Rare/sometimes | Di Fiore (1997); Di Fiore *et al*. (2009); Ellis & Di Fiore (2019) |  |
| *Lemur catta* | yes |  | Gould & Overdorff (2002) |  |
| *Lophocebus albigena* | 24 |  | Brown (2013) |  |
| *Macaca fuscata* |  | 21 | Majolo *et al*. (2005) | Affiliative behaviour was observed in 21% of the encounters but these also contained aggressive elements |
| *Macaca leonina* | yes |  | Choudhury (2008) |  |
| *Macaca mulatta* |  | 7 | Lindburg (1971, 1977) |  |
| *Macaca nemestrina* |  | yes | Bernstein (1967); Oi (1990) |  |
| *Macaca nigra* | 14.8 | 3.1 | Martinez Inigo (2018) |  |
| *Macaca silenus* | 14.3 |  | Kumara *et al*. (2014) |  |
| *Macaca sylvanus* | 7 | yes | Deag & Crook (1971); Deag (1973); Mehlman & Parkhill (1988) |  |
| **Nasalis larvatus* | mostly |  | Bennett & Sebastian (1988); Boonratana (2002); Matsuda *et al*. (2024); Yeager (1991) |  |
| *Pan paniscus* | yes | common | Cheng *et al*. (2022); Fruth & Hohmann (2018); Furuichi (2011, 2020); Idani (1990); Lucchesi *et al*. (2020); Moscovice *et al*. (2022); Sakamaki *et al*. (2018); Sakamaki & Tokuyama (2024); Samuni & Surbeck (2023); Samuni *et al*. (2020); Samuni *et al*. (2024); Tokuyama *et al*. (2019); Tokuyama *et al*. (2021) |  |
| *Pan troglodytes* |  | 42 | Williams *et al*. (2004) | 100% of encounters with adult males on both sides of the interaction were hostile. Only when chimpanzees in the focal community encountered stranger females without males present did peaceful interactions occur. |
| *Papio cynocephalus* |  | yes | Shopland (1982) |  |
| **Papio hamadryas* | common | yes | e.g. Abegglen (1984); Kummer (1968) |  |
| **Papio papio* | common | yes | e.g. Fischer *et al*. (2017) |  |
| *Papio ursinus* | common | 43.5 | Anderson (1981); Cheney & Seyfarth (1977); Cowlishaw (1995) |  |
| *Papio anubis* |  | yes | Packer (1979) |  |
| *Piliocolobus epieni* | 33 |  | Werre (2000) |  |
| *Piliocolobus rufomitratus* | 50 |  | Marsh (1979) |  |
| *Piliocolobus temminckii* | 60 |  | Starin (1991) |  |
| *Piliocolobus tephrosceles* | 72 | yes | Clutton-Brock (1972); Struhsaker (2010) |  |
| *Presbytis potenziani* | common | rare | Fuentes (1994) |  |
| *Propithecus coronatus* | 63 |  | Ramanamisata *et al*. (2014) |  |
| *Propithecus verreauxi* |  | 15 | Lewis *et al*. (2020) |  |
| **Rhinopithecus avunculus* | mostly |  | Boonratana & Le (1994) |  |
| **Rhinopithecus bieti* | mostly | yes | e.g. Grueter *et al*. (2017*a*); Xiang *et al*. (2013) |  |
| **Rhinopithecus brelichi* | mostly |  | Bleisch *et al*. (1993); Nie *et al*. (2009) |  |
| **Rhinopithecus roxellana* | mostly | yes | e.g. Qi *et al*. (2014); Wada *et al.* (2015) |  |
| *Saguinus nigricollis* |  | frequent | De La Torre *et al*. (1995); Izawa (1978) |  |
| *Saguinus fuscicollis* |  | yes | Goldizen (1987) |  |
| *Saimiri sciureus* |  | frequent | Terborgh (1983) |  |
| *Sapajus apella* |  | mostly | Defler (1982) |  |
| *Semnopithecus johnii* |  | yes | Poirier (1968) |  |
| *Simias concolor* | 17 |  | Erb (2012) |  |
| *Symphalangus syndactylus* |  | yes | Palombit (1992) |  |
| **Theropithecus gelada* | common | yes | Dunbar & Dunbar (1975); Mori (1979); Pappano *et al*. (2012) |  |
| *Trachypithecus auratus* | 34.3 |  | Vogt (2003) |  |
| *Trachypithecus crepusculus* |  | 100 | Pengfei Fan, personal communication |  |
| *Trachypithecus geei* | 100 |  | Mukherjee & Saha (1974) |  |
| *Trachypithecus pileatus* |  | yes | Stanford (1991) |  |
| *Semnopithecus entellus* |  | yes | Hrdy (1977); Jay (1965); Yoshiba (1968) |  |

**References**

Abegglen, J. (1984). *On Socialization in Hamadryas Baboons: A Field Study*. Lewisburg: Bucknell University Press.

Anderson, C. M. (1981). Intertroop relations of chacma baboon (*Papio ursinus*). *International Journal of Primatology, 2*(4), 285-310.

Bartlett, T. Q. (2003). Intragroup and intergroup social interactions in white-handed gibbons. *International Journal of Primatology, 24*, 239-259.

Bennett, E. L., & Sebastian, A. C. (1988). Social organization and ecology of proboscis monkeys (*Nasalis larvatus*) in mixed coastal forest in Sarawak. *International Journal of Primatology, 9*, 233-255.

Bermejo, M. (2004). Home-range use and intergroup encounters in western gorillas (*Gorilla g. gorilla*) at Lossi Forest, North Congo. *American Journal of Primatology*, *64*, 223-232.

Bernstein, I. S. (1967). A field study of the pigtail monkey (*Macaca nemestrina*). *Primates, 8*(3), 217-228.

Bleisch, W. V., Cheng, A. S., Ren, X. D., & Xie, J. H. (1993). Preliminary results from a field study of wild Guizhou snub-nosed monkeys (*Rhinopithecus brelichi*). *Folia Primatologica, 60*, 72-82.

Bocian, C. (1997). *Niche Separation of Black-and-White Colobus monkeys (Colobus angolensis and C. guereza) in the Ituri Forest.* (Ph.D. thesis). City University of New York, New York.

Boonratana, R. (2002). Social Organisation of proboscis monkeys (*Nasalis larvatus*) in the Lower Kinabatangan, Sabah, Malaysia *Malayan Nature Journal*(56), 57-75.

Boonratana, R., & Le, X. (1994). *A Report on the Ecology, Status and Conservation of the Tonkin Snub-Nosed Monkey (Rhinopithecus avunculus) in Northern Vietnam*. WCS/IEBR, New York/Hanoi.

Brown, M. (2013). Food and range defence in group-living primates. *Animal Behaviour, 85*(4), 807-816.

Caillaud, D., Levrero, F., Gatti, S., Menard, N., & Raymond, M. (2008). Influence of male morphology on male mating status and behavior during interunit encounters in western lowland gorillas. *American Journal of Physical Anthropology, 135*, 379-388.

Cheney, D. L. (1981). Intergroup encounters among free-ranging vervet monkeys. *Folia Primatologica, 35*(2-3), 124-146.

Cheney, D. L. (1987). Interactions and relationships between groups In B. B. Smuts, D. L. Cheney, R. M. Seyfarth, R. W. Wrangham, & T. T. Struhsaker (Eds.), *Primate Societies* (pp. 267-281). Chicago: University of Chicago Press

Cheney, D. L., & Seyfarth, R. M. (1977). Behaviour of adult and immature male baboons during inter-group encounters. *Nature, 269*(5627), 404-406.

Cheng, L., Samuni, L., Lucchesi, S., Deschner, T., & Surbeck, M. (2022). Love thy neighbour: behavioural and endocrine correlates of male strategies during intergroup encounters in bonobos. *Animal Behaviour, 187*, 319-330.

Choudhury, A. (2008). Ecology and behaviour of the pig-tailed macaque *Macaca nemestrina leonina* in some forests of Assam in North-East India. *Journal of the Bombay Natural History Society, 105*(3), 279-291.

Clutton-Brock, T. (1972). *Feeding and Ranging Behaviour of the Red Colobus Monkey*. PhD thesis, University of Cambridge.

Cooksey, K. E., Sanz, C., Massamba, J. M., Ebombi, T. F., Tebard, P., Magema, E., . . . Morgan, D. (2020). Socioecological factors influencing intergroup encounters in western lowland gorillas (*Gorilla gorilla gorilla*). *International Journal of Primatology*.

Cowlishaw, G. (1995). Behavioural patterns in baboon group encounters: the role of resource competition and male reproductive strategies. *Behaviour, 132*(1-2), 75-86.

De La Torre, S., Campos, F., & De Vries, T. (1995). Home range and birth seasonality of Saguinus nigricollis graellsi in Ecuadorian Amazonia. *American Journal of Primatology, 37*(1), 39-56.

Deag, J., & Crook, J. (1971). Social behaviour and "agonistic buffering" in the wild Barbary macaque Macaca sylvanus L. *Folia Primatologica, 15*, 183-200.

Deag, J. M. (1973). Intergroup encounters in the wild Barbary macaque *Macaca sylvanus* L. In: *Comparative Ecology and Behavior of Primates* (Eds R. P. Michael & J. H. Crook), pp. 315-373. Academic, London.

Decanini, D. P., & Macedo, R. H. (2008). Sociality in *Callithrix penicillata*: II. Individual strategies during intergroup encounters. *International Journal of Primatology, 29*(3), 627-639.

Defler, T. R. (1982). A comparison of intergroup behavior in *Cebus albifrons* and *C. apella*. *Primates, 23*(3), 385-392.

di Fiore, A. (1997). *Ecology and Behavior of Lowland Woolly Monkeys (Lagothrix lagotricha poeppigii, Atelinae) in Eastern Ecuador.* PhD thesis, University of California Davis.

Di Fiore, A., Link, A., Schmitt, C., & Spehar, S. (2009). Dispersal patterns in sympatric woolly and spider monkeys: integrating molecular and observational data. *Behaviour, 146*(4-5), 437-470.

Doran‐Sheehy, D. M., Greer, D., Mongo, P., & Schwindt, D. (2004). Impact of ecological and social factors on ranging in western gorillas. *American Journal of Primatology 64*(2), 207-222.

Dunbar, R. I. M., & Dunbar, E. P. (1975). *Social Dynamics of Gelada Baboons*. Basel, Switzerland: Karger.

Ellis, K., & Di Fiore, A. (2019). Variation in space use and social cohesion within and between four groups of woolly monkeys (*Lagothrix lagotricha poeppigii*) in relation to fruit availability and mating opportunities at the Tiputini Biodiversity Station, Ecuador. In: *Movement Ecology of Neotropical Forest Mammals* (Eds R. Reyna-Hurtado & C. Chapman), pp. 141-171. Springer, Cham, Switzerland.

Erb, W. M. (2012). *Male-male Competition and Loud Calls in One-male Groups of Simakobu (Simias concolor).* PhD thesis, State University of New York at Stony Brook.

Fischer, J., Kopp, G. H., Dal Pesco, F., Goffe, A., Hammerschmidt, K., Kalbitzer, U., . . . Zinner, D. (2017). Charting the neglected West: The social system of Guinea baboons. *American Journal of Physical Anthropology 162*, 15-31.

Forcina, G., Vallet, D., Le Gouar, P. J., Bernardo-Madrid, R., Illera, G., Molina-Vacas, G., . . . Bermejo, M. (2019). From groups to communities in western lowland gorillas. *Proceedings of the Royal Society B: Biological Sciences, 286*, 20182019.

Fruth, B., & Hohmann, G. (2018). Food sharing across borders: first observation of intercommunity meat sharing by bonobos at LuiKotale, DRC. *Human Nature, 29*, 91-103.

Fuentes, A. (1994). *The Socioecology of the Menawai Island Langur.* PhD thesis, University of California, Berkeley.

Furuichi, T. (2011). Female contributions to the peaceful nature of bonobo society. *Evolutionary Anhropology, 20*, 131-142.

Furuichi, T. (2020). Variation in intergroup relationships among species and among and within local populations of African apes. *International Journal of Primatology*.

Gautier-Hion, A., & Gautier, J. (1978). Le singe de Brazza: une stratégie originale. *Zeitschrift fuer Tierpsychologie* 46, 84-104.

Goldizen, A. W. (1987). Tamarins and marmosets: communal care of offspring. In: *Primate Societies* (Eds B. B. Smuts, D. L. Cheney, R. M. Seyfarth, R. W. Wrangham, T. T. Struhsaker), pp. 34-43. University of Chicago Press, London.

Gould, L., & Overdorff, D. J. (2002). Adult male scent-marking in *Lemur catta* and *Eulemur fulvus rufus*. *International Journal of Primatology, 23*(3), 575-586.

Greenway, K. (2015). *Threat and Display: Reproductive Competition in Wild Male Western Gorillas (Gorilla gorilla).* PhD thesis, University of Kent.

Grueter, C. C., Li, D., Ren, B., Wei, F., & Li, M. (2017*a*). Deciphering the social organization and structure of wild Yunnan snub-nosed monkeys (*Rhinopithecus bieti*). *Folia Primatologica, 88*, 358-383.

Hrdy, S. (1977). *The Langurs of Abu: Female and Male Strategies of Reproduction*. Harvard University Press, Cambridge.

Idani, G. (1990). Relations between unit-groups of bonobos at Wamba, Zaire: encounters and temporary fusions. *African Study Monographs, 11*, 153-186.

Izawa, K. (1978). A field study of the ecology and behavior of the black-mantle tamarin (*Saguinus nigricollis*). *Primates, 19*, 241-274.

Jay, P. (1965). The common langur of north India. In *Primate Behavior: Field Studies of Monkeys and Apes* (Ed I. DeVore), pp. 197-249. Holt, Rinehart & Winston, New York.

Kinnaird, M. F. (1992). Variable resource defense by the Tana River crested mangabey *Behavioral Ecology and Sociobiology, 31*, 115-122

Korstjens, A., Nijssen, E., & Noë, R. (2005). Intergroup relationships in western black-and-white colobus, *Colobus polykomos polykomos*. *International Journal of Primatology, 26*, 1267-1289

Korstjens, A. H. (2001). *The Mob, the Secret Sorority, and the Phantoms.* PhD thesis, Utrech University, Utrecht.

Kowalewski, M. M. (2007). *Patterns of Affiliation and Co-operation in Howler Monkeys: an Alternative Model to Explain Social Organization in Non-human Primates.* PhD thesis, University of Illinois at Urbana-Champaign,

Kumara, H. N., Singh, M., Sharma, A. K., Santhosh, K., & Pal, A. (2014). Impact of forest fragment size on between-group encounters in lion-tailed macaques. *Primates, 55*(4), 543-548.

Kummer, H. (1968). *Social Organization of Hamadryas Baboons: A Field Study*. Chicago: The University of Chicago Press.

Lawes, M. J., & Henzi, S. P. (1995). Inter-group encounters in blue monkeys: how territorial must a territorial species be? *Animal Behaviour* 49(1), 240-243.

Lazaro-Perea, C. (2001). Intergroups interactions in wild common marmosets, *Callithrix jacchus*: territorial defence and assessment of neighbours. *Animal Behaviour, 62*, 11-21.

Lewis, R. J., Sandel, A. A., Hilty, S., & Barnett, S. E. (2020). The collective action problem but not numerical superiority explains success in intergroup encounters in Verreaux's sifaka (*Propithecus verreauxi*): implications for individual participation and free-riding. *International Journal of Primatology, 41*, 305-324.

Lindburg, D. G. (1971). The rhesus monkey in north India: an ecological and behavioral study. In *Primate Behavior: Developments in Field and Laboratory Research. Volume 2* (Ed L. A. Rosenblum), pp. 1-106. Academic Press, New York.

Lindburg, D. G. (1977). Feeding behaviour and diet of rhesus monkeys (*Macaca mulatta*) in a Siwalik forest in North India. In *Primate Ecology: Studies of Feeding and Ranging Behaviour in Lemurs, Monkeys and Apes* (Ed T. H. Clutton-Brock), pp. 223-249. Academic Press, New York.

Lucchesi, S., Cheng, L., Janmaat, K., Mundry, R., Pisor, A., & Surbeck, M. (2020). Beyond the group: how food, mates, and group size influence intergroup encounters in wild bonobos. *Behavioral Ecology, 31,* 519-532.

Magliocca, F., & Gautier-Hion, A. (2004). Inter-group encounters in western lowland gorillas at a forest clearing. *Folia Primatologica, 75*(6), 379-382.

Majolo, B., Ventura, R., & Koyama, N. F. (2005). Sex, rank and age differences in the Japanese macaque (*Macaca fuscata yakui*) participation in inter‐group encounters. *Ethology, 111*(5), 455-468.

Marsh, C. (1979). Comparative aspects of social organization in the Tana River red colobus, Colobus badius rufomitratus. *Zeitschrift fuer Tierpsychologie 51*, 337-362.

Martinez Inigo, L. (2018). *Intergroup Interactions in Crested Macaques (Macaca nigra): Factors Affecting Intergroup Encounter Outcome and Intensity.* PhD thesis, University of Lincoln,

Matsuda, I., Murai, T., Grueter, C. C., Tuuga, A., Goossens, B., Bernard, H., . . . Salgado-Lynn, M. (2024). The multilevel society of proboscis monkeys with a possible patrilineal basis. *Behavioral Ecology and Sociobiology, 78*(1), 5.

Mehlman, P. T., & Parkhill, R. S. (1988). Intergroup interactions in wild barbary macaques (*Macaca sylvanus*), Ghomaran Rif Mountains, Morocco. *American Journal of Primatology, 15*(1), 31-44.

Miller, A., Uddin, S., Judge, D. S., Kaplin, B., Ndayishimiye, D., Uwingeneye, G., & Grueter, C. C. (2020). Spatiotemporal association patterns in a supergroup of Rwenzori black‐and‐white colobus (*Colobus angolensis ruwenzorii*) are consistent with a multilevel society. *American Journal of Primatology* *82*, e23127.

Mirville, M. O., Ridley, A. R., Samedi, J. P. M., Vecellio, V., Ndagijimana, F., Stoinski, T. S., & Grueter, C. C. (2018). Low familiarity and similar ‘group strength’ between opponents increase the intensity of intergroup interactions in mountain gorillas (*Gorilla beringei beringei*). *Behavioral Ecology and Sociobiology, 72*, 178.

Mori, U. (1979). Inter-unit relationships. In: Ecological and Sociological Studies of Gelada Baboons. *Contributions to Primatology* *16*, 83–92.

Moscovice, L. R., Hohmann, G., Trumble, B. C., Fruth, B., & Jaeggi, A. V. (2022). Dominance or tolerance? Causes and consequences of a period of increased intercommunity encounters among bonobos (*Pan paniscus*) at LuiKotale. *International Journal of Primatology*, *43*(3), 434-459.

Mukherjee, R. P., & Saha, S. S. (1974). The golden langurs (*Presbytis geei* Khajuria, 1956) of Assam. *Primates, 15*, 327-340.

Nie, S., Xiang, Z., & Li, M. (2009). Preliminary report on the diet and social structure of gray snub-nosed monkeys (*Rhinopithecus brelichi*) at Yangaoping,Guizhou,China. *Acta Theriologica Sinica, 29*, 326-331.

Nievergelt, C. M., Mutschler, T., & Feistner, A. T. C. (1998). Group encounters and territoriality in wild Alaotran gentle lemurs (*Hapalemur griseus alaotrensis*). *American Journal of Primatology, 46*(3), 251-258.

Oi, T. (1990). Population organization of wild pig-tailed macaques (*Macaca nemestrina nemestrina*) in West Sumatra. *Primates, 31*(1), 15-31.

Packer, C. (1979). Inter-troop transfer and inbreeding avoidance in *Papio anubis Animal Behaviour, 27*, 1-36

Palombit, R. A. (1992). *Pair bonds and monogamy in wild siamang (Hylobates syndactylus) and white-handed gibbon (Hylobates lar) in northern Sumatra*: University of California, Davis.

Pappano, D. J., Snyder-Mackler, N., Bergman, T. J., & Beehner, J. C. (2012). Social ‘predators’ within a multilevel primate society. *Animal Behaviour, 84*, 653-658.

Parnell, R. J. (2002). *The social structure and behaviour of western lowland gorillas (Gorilla gorilla gorilla) at Mbeli Bai, Republic of Congo*. PhD thesis, University of Stirling.

Pinto, A. C. B., Azevedo-Ramos, C., & de Carvalho Jr, O. (2003). Activity patterns and diet of the howler monkey *Alouatta belzebul* in areas of logged and unlogged forest in Eastern Amazonia. *Animal Biodiversity and Conservation, 26*(2), 39-49.

Poirier, F. E. (1968). Nilgiri langur (*Presbytis johnii*) territorial behavior. *Primates, 9*, 351-364.

Powzyk, J. A. (1997). *The socio-ecology of two sympatric Indrids. Propithecus diadema diadema and Indri indri: A comparison of feeding strategies and their possible repercussions on species-specific behaviors*. PhD thesis, Duke University.

Ramanamisata, R., Pichon, C., Razafindraibe, H., & Simmen, B. (2014). Social behavior and dominance of the crowned sifaka (*Propithecus coronatus*) in northwestern Madagascar. *Primate Conservation, 28*, 93-97.

Sakamaki, T., Ryu, H., Toda, K., Tokuyama, N., & Furuichi, T. (2018). Increased frequency of intergroup encounters in wild bonobos (*Pan paniscus*) around the yearly peak in fruit abundance at Wamba. *International Journal of Primatology, 39*, 685-704.

Sakamaki, T., & Tokuyama, N. (2024). Potential benefits of intergroup associations and chronological changes of intergroup relationships in bonobos. In *Bonobos and People at Wamba: 50 Years of Research* (Eds T. Furuichi, G. Idani, D. Kimura, H. Ihobe, C. Hashimoto), pp. 311-332. Springer, Singapore.

Samuni, L., & Surbeck, M. (2023). Cooperation across social borders in bonobos. *Science, 382*(6672), 805-809.

Samuni, L., Wegdell, F., & Surbeck, M. (2020). Behavioural diversity of bonobo prey preference as a potential cultural trait. *Elife, 9*, e59191.

Samuni, L., Wessling, E. G., & Surbeck, M. (2024). Rethinking peace from a bonobo perspective. *Behavioral and Brain Sciences, 47*, e27.

Sato, H. (2013). Habitat shifting by the common brown lemur (*Eulemur fulvus fulvus*): a response to food scarcity. *Primates, 54*(3), 229-235.

Shopland, J. M. (1982). An intergroup encounter with fatal consequences in yellow baboons (*Papio cynocephalus*). *American Journal of Primatology, 3*(1‐4), 263-266.

Sicotte, P. (1993). Inter-group encounters and female transfer in mountain gorillas: Influence of group composition on male behavior. *American Journal of Primatology, 30(1)*, 21-36.

Stanford, C. (1991). Social dynamics of of intergroup encounters in the capped langur (*Presbytis pileata*). *American Journal of Primatology, 25*, 35-47.

Starin, E. D. (1991). *Socioecology of the Red Colobus Monkey in the Gambia with Particular Reference o Female-Male Differences and Transfer Patterns.* PhD thesis, City University of New York, New York.

Stead, S. M., & Teichroeb, J. A. (2019). A multi-level society comprised of one-male and multi-male core units in an African colobine (*Colobus angolensis ruwenzorii*). *Plos One* *10*, e0217666.

Struhsaker, T. T. (2010). *The Red Colobus Monkeys: Variation in Demography, Behavior, and Ecology of Endangered Species*. Oxford University Press, Oxford.

Suwanvecho, U., & Brockelman, W. Y. (2012). Interspecific territoriality in gibbons (*Hylobates lar* and *H. pileatus*) and its effects on the dynamics of interspecies contact zones. *Primates, 53*(1), 97-108.

Teichroeb, J. A., & Sicotte, P. (2018). Cascading competition: the seasonal strength of scramble influences between-group contest in a folivorous primate. *Behavioral Ecology and Sociobiology, 72*(1), 1-16.

Tenaza, R. R. (1975). Territory and monogamy among Kloss’ gibbons (*Hylobates klossii*) in Siberut Island, Indonesia. *Folia Primatologica, 24*(1), 60-80.

Terborgh, J. (1983). *Five New World Primates*: *A Study in Comparative Ecology* Princeton: Princeton University Press.

Tokuyama, N., Sakamaki, T., & Furuichi, T. (2019). Inter‐group aggressive interaction patterns indicate male mate defense and female cooperation across bonobo groups at Wamba, Democratic Republic of the Congo. *American Journal of Physical Anthropology, 170*(4), 535-550.

Tokuyama, N., Toda, K., Poiret, M.-L., Iyokango, B., Bakaa, B., & Ishizuka, S. (2021). Two wild female bonobos adopted infants from a different social group at Wamba. *Scientific Reports, 11*(1), 4967.

Vogt, M. (2003). *Freilanduntersuchungen zur Ökologie und zum Verhalten von Trachypithecus auratus kohlbruggei (Haubenlanguren) im West-Bali-Nationalpark, Indonesien.* Eberhard-Karls-Universität, Tübingen.

Werre, J. (2000). *Ecology and Behavior of the Niger Delta Red Colobus (Procolobus badius epieni).* (PhD). City University, New York.

Williams, J. M., Oehlert, G. W., Carlis, J. V., & Pusey, A. E. (2004). Why do male chimpanzees defend a group range? *Animal Behaviour, 68*(3), 523-532.

Xiang, Z., Xiao, W., Huo, S., & Li, M. (2013). Ranging pattern and population composition of *Rhinopithecus bieti* at Xiaochangdu: Implications for conservation. *Chinese Science Bulletin, 58*, 2212.

Yeager, C. P. (1991). Proboscis monkey (*Nasalis larvatus*) social organization: Intergroup patterns of association. *American Journal of Primatology, 23*, 73-86.

Yoshiba, K. (1968). Local and intergroup variability in ecology and social behavior of common Indian langurs. *Primates: Studies in adaptation and variability*.
